# Rehabilitation among individuals experiencing homelessness and traumatic brain injury: A scoping review

**DOI:** 10.3389/fmed.2022.916602

**Published:** 2022-11-11

**Authors:** Vincy Chan, Maria Jennifer Estrella, Richelle Baddeliyanage, Riya Shah, Jessica Babineau, Angela Colantonio

**Affiliations:** ^1^KITE Research Institute, Toronto Rehabilitation Institute-University Health Network, Toronto, ON, Canada; ^2^Rehabilitation Sciences Institute, University of Toronto, Toronto, ON, Canada; ^3^Institute of Health Policy, Management and Evaluation, University of Toronto, Toronto, ON, Canada; ^4^Department of Occupational Science and Occupational Therapy, University of Toronto, Toronto, ON, Canada; ^5^Dalla Lana School of Public Health, University of Toronto, Toronto, ON, Canada; ^6^Library and Information Services, University Health Network, Toronto, ON, Canada; ^7^The Institute for Education Research, University Health Network, Toronto, ON, Canada

**Keywords:** rehabilitation, occupational therapy, homeless persons, brain injuries, cognitive impairment, public health, diversity

## Abstract

Traumatic brain injury (TBI) is disproportionately prevalent among individuals experiencing homelessness. While rehabilitation is critical to facilitating recovery after TBI, there is currently limited information on the extent to which rehabilitation is provided to individuals experiencing homelessness and TBI. If unaddressed, this knowledge gap can perpetuate TBI-related challenges and contribute to a repetitive cycle of TBI and homelessness. This scoping review explored the extent to which rehabilitation, including the types of rehabilitation interventions, are available to, or used by, individuals experiencing homelessness and TBI. A systematic search of electronic databases (MEDLINE, Embase, Cochrane CENTRAL Register of Clinical Trials, CINAHL, APA PsycINFO, Applied Social Sciences Index and Abstracts, and Proquest Nursing and Allied Health) was conducted to identify peer-reviewed articles that met predetermined eligibility criteria. Gray literature and reference lists of eligible articles were also searched for relevant content. A descriptive numerical summary of extracted data was conducted, and qualitative analytic techniques were applied to analyze the data. Fifteen peer-reviewed articles and three gray literature reports were included, describing interventions for individuals experiencing homelessness and TBI (*N* = 4), rehabilitation for individuals experiencing homelessness without specific inclusion criteria for TBI (*N* = 11), and rehabilitation interventions that included individuals experiencing homelessness and TBI, without specific inclusion criteria for experiences of homelessness or TBI (*N* = 3). This review demonstrates that rehabilitation programs or interventions for this population already exist, and those that are focused on individuals experiencing homelessness are already serving individuals with TBI. Findings highlight opportunities to adapt existing rehabilitation for individuals who experience homelessness and TBI through screening for TBI, conducting cognitive and functional assessments, and tailoring interventions with multidisciplinary teams. Education and training for healthcare professionals working with individuals experiencing homelessness and TBI should be explored, including structured education and training, collaboration with a multidisciplinary team, and co-development of educational materials with service users. Research that considers the rehabilitation needs of diverse individuals experiencing homelessness and TBI is urgently needed.

## Introduction

Homelessness is a serious public health concern facing modern society. An estimated 580,466 individuals in the United States experienced homelessness in 2020 (1) and based on the most recent reported national Point-in-Time count, an average of at least 235,000 individuals in Canada are experiencing homelessness in every year (2). Globally, one in five individuals experience housing insecurity (3). Homelessness arises from a complex interplay of structural and individual factors, and is associated with a broad range of health conditions (4, 5) such as infections (e.g., tuberculosis and HIV), cardiovascular and respiratory conditions, and psychiatric and substance use disorders (SUD) (6, 7). Together, these factors lead to substantially high rates of premature mortality compared to the general population (6, 7). Cognitive and functional impairments are also prevalent (8, 9), and recent evidence has identified traumatic brain injury (TBI) as a determinant of cognitive and neurological disability in the homeless population (10, 11).

TBI, defined as “an alteration in brain function or other evidence of brain pathology caused by an external force” (12), is a leading cause of death and disability worldwide that is under-recognized (13), highly prevalent, and can cause life-long debilitating challenges among individuals experiencing homelessness (14–16). A recent systematic review estimated that the lifetime prevalence of TBI of any severity was 53.1% among homeless and vulnerably housed persons (17). While there is no causal link, the relationship between TBI and homelessness is considered bidirectional, wherein experiencing homelessness could increase an individual's vulnerability to TBI and having a TBI could increase the risk for experiencing homelessness (10, 17). TBI is associated with poorer self-reported physical and mental health and suicidality, challenges in memory, greater use of health services, involvement in the criminal justice system (15, 18), and a longer duration of homelessness and housing instability (15). The challenges associated with TBI are exacerbated by individual and structural factors and intersecting social inequities that without intervention could intensify and lead to a repetitive cycle of TBI, homelessness, and significant health-related costs (4, 10, 16, 18, 19).

Rehabilitation, defined as “a set of interventions designed to optimize functioning and reduce disability in individuals with health conditions in interaction with their environment,” (20) is considered a critical component of TBI management (21, 22). In the context of TBI, rehabilitation ranges from early clinical management that focuses on immediate treatment needs post-injury, to ongoing therapeutic and pharmacological interventions that target long-term functional and cognitive impairments (23, 24). Such rehabilitation interventions have demonstrated positive effects in addressing TBI sequelae, promoting functional recovery, and improving quality of life (23, 25, 26) and are well-documented in evidence-based clinical practice guidelines that guide TBI care (27). However, despite recent guidelines and reviews on TBI rehabilitation (24, 27–29) or homelessness (15, 30–32), to the best of our knowledge, no review to date has focused on rehabilitation for individuals experiencing homelessness and TBI.

This scoping review responds to this gap by exploring the extent to which rehabilitation, including the types of rehabilitation interventions, are available to, or used by, individuals experiencing homelessness and TBI. This review also explicitly charts and summarizes evidence on age, sex, gender, ethnicity, race, and other identities and experiences, as individuals experiencing homelessness also experience health disparities that are shaped by their intersecting identities (33). The results of this scoping review inform (a) opportunities to adapt existing rehabilitation for individuals who experience homelessness and TBI, (b) considerations for education and training on TBI, and (c) recommendations for future research. Overall, this scoping review provides the foundation for advancing rehabilitation for individuals experiencing homelessness and TBI.

## Methods and analysis

This scoping review was guided by methodology frameworks from Arksey and O'Malley (34) and Levac et al. (35) and the reporting of this scoping review follows the Preferred Reporting Items for Systematic Reviews and Meta-Analyses extension for Scoping Reviews (PRISMA-ScR) (36). The protocol for this scoping review is published in the journal *BMJ Open* (37) and is summarized below.

### Stage 1: Identifying the research question

The research question this scoping review answered was “To what extent is rehabilitation, including the types of rehabilitation interventions, available to, or used by, individuals experiencing homelessness and TBI?” The definitions of rehabilitation and homelessness that were used to guide the scoping review is presented in Table 1.

**Table 1:** Definitions of rehabilitation and homelessness.

**Concept**	**Definitions**
Rehabilitation	World Health Organization—“A set of interventions designed to optimize functioning and reduce disability in individuals with health conditions in interaction with their environment” (20) Healthcare providers/professional disciplines identified in TBI evidence-based clinical practice guidelines for TBI rehabilitation (27, 29): • Neuropsychologist • Nurse • Nutritionist • Occupational therapist • Physiatrist • Physician • Physiotherapist • Psychologist with expertise in behavioral therapy • Psychometrist • Rehabilitation support personnel • Social worker • Speech-language pathologist • Therapeutic recreationist
Homelessness	Canadian Observatory of Homelessness' typology of homelessness that encompasses the following physical living situations at the time of the research study (38): • Unsheltered—individuals who lack housing and are not accessing shelters • Emergency sheltered—individuals who cannot secure permanent housing and are accessing shelters or other system supports • Provisionally accommodated—individuals without permanent shelter and are accessing accommodations that offer no prospect of permanent

### Stage 2: Identifying the relevant studies

The search strategy was developed in collaboration with an Information Specialist (JB) and was conducted in the following databases: MEDLINE ALL (Ovid), Embase and Embase Classic (Ovid), Cochrane CENTRAL Register of Clinical Trials (Ovid), CINAHL (EBSCO), APA PsycINFO (Ovid), Applied Social Sciences Index and Abstracts (Proquest), and Nursing and Allied Health (Proquest). The search strategy was first conducted in April 2021 and updated in March 2022 with no changes to the strategy. Three concepts—(A) homelessness, (B) rehabilitation, and (C) TBI or cognitive impairment—were used to develop the search strategy of (A + B) OR (A + C). In addition to searching databases, reference lists of included articles were also searched. Gray literature, defined in this review as reports from relevant brain injury, housing, or rehabilitation organizations, were searched for relevant content in between May 2021 and September 2021. Specifically, they were searched by entering keywords for concepts A, B, and C in the search bar. Websites without a search bar were manually reviewed for relevant gray literature reports. No limits on language or dates were placed on the search. Supplementary File 1 presents the search strategy for databases and websites of brain injury, housing, and rehabilitation organizations that were searched for gray literature.

### Stage 3: Study selection

Eligible studies were peer-reviewed primary research articles or gray literature that met the following criteria: (1) describe and/or document rehabilitation interventions or describe and/or document services provided by healthcare providers or professional disciplines, as defined in Table 1; (2) focus on individuals who are experiencing homelessness at the time of the research study, as defined in Table 1; and (3) include individuals with TBI. The following articles were excluded: (1) dissertations, conference proceedings, and articles that are narrative, commentaries or describe a theory of framework without reporting primary research findings and (2) articles that include the broader brain-injured population without specific mention of TBI (e.g., individuals with acquired brain injury, cognitive impairment).

EndNote X8.2 was used for reference management (39) and Covidence was used for de-duplication and study selection (40). Two reviewers (RB and RS for the search conducted in April 2021 and VC and MJE for the search updated in March 2022) independently screened all articles based on the above pre-determined eligibility criteria. At the title and abstract screen, scoping, and systematic reviews that met the above eligibility criteria and articles that did not explicitly mention TBI were also included for full-text review. Non-English language abstracts were assessed using the published English abstract. A pilot screen of 20 titles and abstracts was conducted until a minimum 80% agreement was achieved between the two reviewers. The resulting agreement at the title and abstract screen was 85.2% (RB and RS) and 97.6% (VC and MJE) for English language articles and 89.7% (RB and RS) and 80.0% (VC and MJE) for non-English language articles. Discrepancies were resolved by consensus or consultation with a third reviewer (VC or MJE).

At the full-text screen, two reviewers (RB and RS for the search conducted in April 2021 and VC and MJE for the search updated in March 2022) independently screened all articles based on the above eligibility criteria. For scoping and systematic reviews identified in the title and abstract screen, the primary research articles included in the reviews were extracted and screened according to the above eligibility criteria. Non-English language articles were translated to English language using Google Translate (41) and/or DeepL Translate (42). A pilot screen of 10% of eligible full-text articles was conducted until a minimum of 80% agreement was achieved between the two reviewers. The resulting agreement at the full-text screen was 97.6% (RB and RS; VC and MJE) for English language articles and 89.7% (RB and RS) and 100% (VC and MJE) for non-English language articles. Discrepancies were resolved by consensus or consultation with a third reviewer (VC or MJE).

### Stage 4: Charting the data

The charting table was completed independently by one reviewer (RB or VC) and peer-reviewed by two reviewers (RS and/or VC). The resulting charting table is presented in Supplementary File 2 and was used to inform Stage 5 of the scoping review. Discrepancies in charting the data were resolved by consensus or consultation with a third reviewer (VC or MJE).

### Stage 5: Collating, summarizing, and reporting the results

Three distinct steps, as outlined by Levac's et al. methodology framework (35), were followed: (1) analyzing the data, (2) reporting the results, and (3) applying meaning to the results. To analyze the data, a descriptive numerical summary of the data extracted and presented in the charting table was compiled and qualitative content analytic techniques were applied to allow for the quantification of data in themes or category development. The results were reported in relation to the research question, using findings from the data analyses. To apply meaning to the results, implications for (a) opportunities to adapt existing rehabilitation for individuals who experience homelessness and TBI, (b) considerations for education and training on TBI, and (c) recommendations for future research were considered.

Quality appraisal, although not specified in the methodology frameworks, was conducted by one reviewer (VC) and peer-reviewed by a second reviewer (RS). The Study Quality Assessment Tools designed by methodologists from the Research Triangle Institute International and the National heart, Lung, and Blood Institute of the National Institutes of Health and the Critical Appraisal Skills Programme (43) were used to inform the internal validity of a variety of study designs. No articles were eliminated based on the quality assessment; however, findings were used to inform the process of applying meaning to the study and to identify opportunities to advance research on rehabilitation among individuals experiencing homelessness and TBI.

### Stage 6: Consultation

Preliminary findings from Stage 5 were shared with the Program Advisory Committee (PAC) of the Traumatic Brain Injury in Underserved Populations Research Program (44). This PAC consists of service providers across brain injury, disability, housing, criminal justice, and violence against women sectors. Specifically, preliminary findings were presented at a PAC meeting and PAC members' feedback were incorporated in the discussion of this review.

## Results

**Figure 1:**
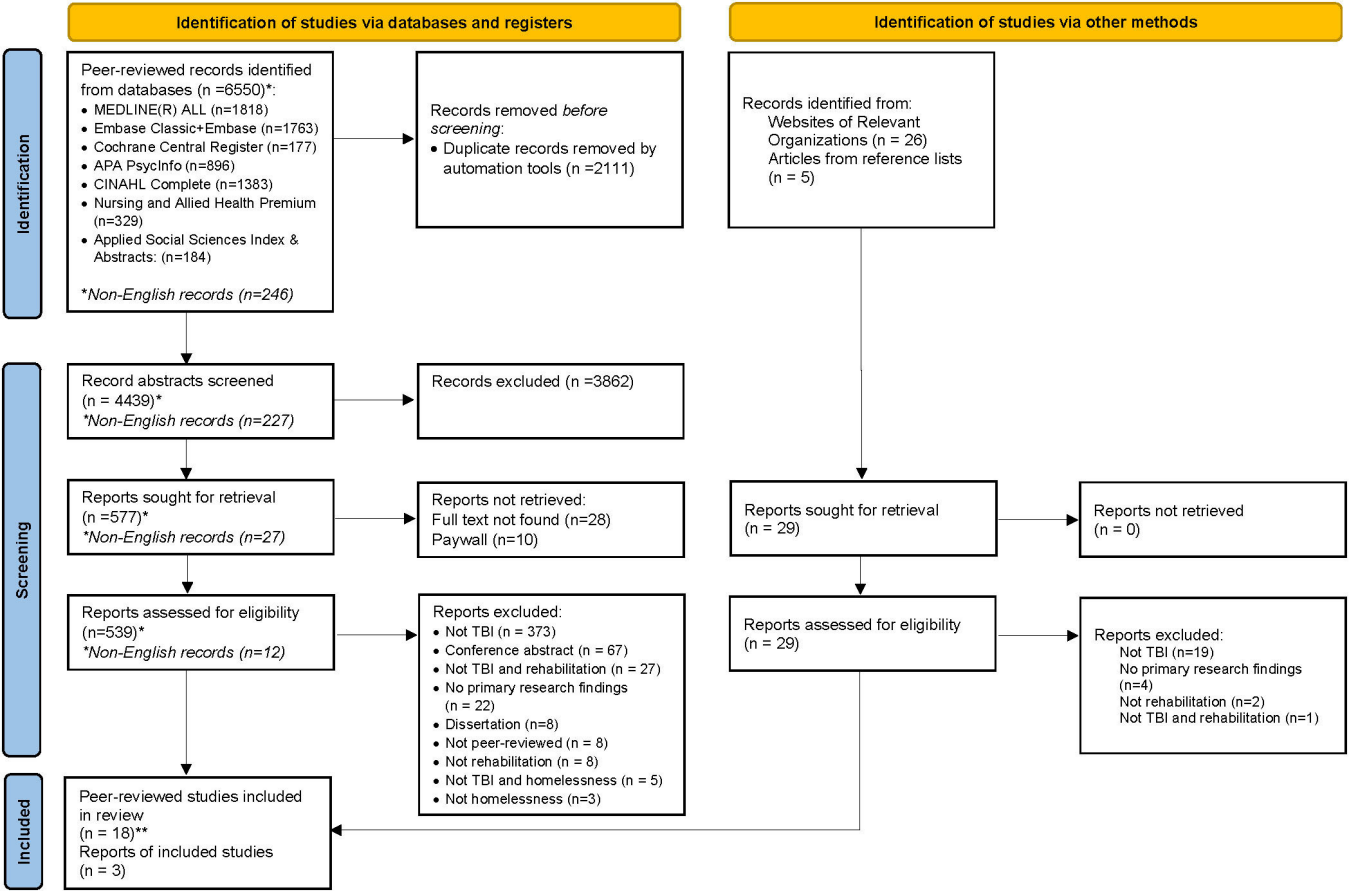
PRISMA 2020 flow diagram for new systematic reviews which included searches of databases, registers, and other sources.

A total of 6,550 citations were identified from databases through the search strategy. After duplicates were removed, 4,439 titles and abstracts (227 non-English language) were screened, of which 577 (27 non-English language) met eligibility for full-text review. Of these articles, 532 were excluded based on the pre-determined eligibility criteria, resulting in 15 primary research articles included in this synthesis. Gray literature search identified an additional 3 reports. Figure 1 presents the PRISMA flow diagram for the search and screening.

All the articles identified through the search described research conducted in the United States (*N* = 10, 55.6%) (45–54) or Canada (*N* = 8, 44.4%) (55–62). Four articles (22.2%) described interventions for individuals experiencing homelessness and TBI (46, 51, 57, 59). The remaining articles described rehabilitation interventions for individuals experiencing homelessness without specific inclusion criteria for TBI (*N* = 11, 61.1%) (45, 49, 50, 52, 54–56, 58, 60–62) or rehabilitation interventions that included individuals experiencing homelessness and TBI, without specific inclusion criteria for experiences of homelessness or TBI (*N* = 3, 16.7%) (47, 48, 53). None of the rehabilitation programs or interventions were based at an inpatient or outpatient rehabilitation setting; 17 of 18 articles described community-based rehabilitation that were offered through community organization healthcare clinics (45, 47–62) or mobile clinic (54), while one article described a medical respite program that provided care to individuals onsite (46).

Males or men comprised the majority of the study sample in 11 articles, ranging from 52 to 90% (45, 48, 52, 54–62), and one article included only women in their sample (47). Except for case studies (*N* = 2) (46, 53), none of the studies stratified the findings by sex or gender. In articles that did not explicitly focus on individuals experiencing both homelessness and TBI, the proportion of individuals with TBI ranged from 2.4 to 84% and the proportion of individuals experiencing homelessness ranged from 1.2 to 100%. The majority of articles did not define how TBI was ascertained, with the exception of three articles that screened for self-reported TBI using the Ohio State University Traumatic Brain Injury Identification Method (OSU-TBI-ID) (46, 51, 57) and one article through face-to-face interviews (59). None of the articles reported injury severity.

Fourteen articles defined homelessness as part of their inclusion criteria, description of the study sample, or study setting [“absolutely or precariously housed” (55, 56, 58, 59), “experiencing homelessness” (45), “doubling up—friends and family” (62), “shelter, unsheltered, doubled-up” (54) “residing in homeless shelter or on the street” (47, 48, 51), “scattered site apartments” (45), “respite program” (46), “transitional housing” (62) or participants were referred to as “homeless individuals” (49, 50, 52, 53, 57)]. Six articles reported participants intersected with the justice system [i.e., “ever been to jail” (45), “ever been to prison” (45), “arrested in the past” (58), “arrests in the past 6 months” (56), “times in jail/prison” (49), “had prior justice involvement” (60, 61)], with prevalence ranging from 16 to 80% (45, 49, 56, 58, 60, 61). Table 2 presents key study characteristics, Supplementary File 2 presents the charting table, and Supplementary File 3 presents the quality appraisal of included articles.

**Table 2:** Study characteristics and description of rehabilitation.

**Study characteristics**	***N***
**Country of study**	
United States (45–54)	10
Canada (55–62)	8
**Study design** ^**a**^	
Controlled interventions (55, 56, 58–61)	6
Observational cohort/cross-sectional (48, 49, 51–54, 57, 62)	8
Case studies/series (46, 53)	2
Before-After no control groups (45, 47)	2
Qualitative (50)	2
**Age eligibility for rehabilitation program/intervention**	
Adults (≥18 years) (51, 52, 54–56, 58–61)	9
Adults only (18–65 years) (48)	1
Not reported^b^ (45–47, 49, 50, 53, 57, 62)	8
**Sex/Gender**	
Data stratified by sex or gender^c^ (46, 53)	2
Females/women only (47)	1
Authors' reference to sex and gender:	
a) Sex: Males/females (49, 54)	2
b) Gender: Men/women (59)	1
c) Gender: Males/females (45, 48, 55, 56, 58, 60–62)	8
d) No reference to sex or gender but used the terms males/females (52, 53, 57)	3
e) No reference to sex or gender but used the terms men/women (47)	1
f) Not reported (46, 50, 51)	3
**Description of rehabilitation program/intervention**	
**Target population**	
Individuals experiencing homelessness and TBI (46, 51, 57, 59)	4
Individuals experiencing homelessness without specific inclusion criteria for TBI (45, 49, 50, 52, 54–56, 58, 60–62)	11
Rehabilitation without specific inclusion criteria for experiences of homelessness or TBI (47, 48, 53)	3
**Location of rehabilitation program/intervention**	
Community-Based rehabilitation offered through:	
a) Community organizations (45, 47–49, 53, 55–62)	13
b) Healthcare clinics (50–52)	3
c) Mobile clinic (54)	1
Medical respite program (onsite care) (46)	1

TBI, Traumatic brain injury.

^a^One article described an observational cohort/cross sectional and case study and one report described an observational cohort/cross sectional and qualitative study.

^b^While the age eligibility of the rehabilitation program or intervention was not reported, the average age and/or age range was reported in these articles.

^c^All case studies.

### Qualitative content analysis of the three categories of articles

Findings from the three categories of rehabilitation articles identified in this scoping review are described below. Within each of the study categories, rehabilitation in the form of multidisciplinary care and specialized care were identified. For this review, multidisciplinary care refers to articles describing rehabilitation provided by more than one type of healthcare provider/professional discipline, whereas specialized care refers to articles describing rehabilitation provided by a specific healthcare provider/professional discipline.

#### Rehabilitation program and interventions for individuals experiencing homelessness and TBI (N = 4)

All four articles reported rehabilitation within a multidisciplinary care context, with services accessible through a multidisciplinary team of professionals and/or a case manager. Brocht et al. described a shelter-based respite program that offered access to healthcare professionals (i.e., on-site occupational therapists (OTs), social workers, registered nurses, and community health workers) (46). They detailed the roles of different healthcare professionals that contributed to the creation of a TBI-focused program. Social workers or OTs screened for TBI using the OSU-TBI-ID. Following TBI screening, OTs assessed both the cognitive and functional abilities of clients in their living environment through standardized cognitive assessment tools and functional evaluations. OTs then educated respite staff regarding TBI-related functional limitations and behaviors and collaborated with them regarding concrete strategies to manage such limitations and behaviors (e.g., external cueing strategies and environmental modifications). Other members of the respite centre's multidisciplinary team reinforced OTs' recommendations while performing specialized roles. Social workers supported discharge planning, case management, and provided psychotherapy evaluation, brief intervention, and linkage to housing. Registered nurses provided health education, coordinated specialty follow-up, and interpreted information from medical provider to patients. Community health workers implemented treatment plans developed by the OTs and provided supports to patients, both direct (e.g., transportation, paperwork) and relational (e.g., maintaining positive recovery environment, developing rapport with patients). Synovec and Berry described a study on screening and provider training at a healthcare clinic (51). Comprehensive care in terms of services, such as medical, mental health, case management, occupational therapy, dental services, and supportive housing were provided. While the roles of each healthcare provider were not delineated in this study, the importance of TBI screening and strategies to address TBI-related limitations were emphasized.

Two articles described assertive community treatment (ACT) as an intervention (57, 59); however, one was specifically delivered in the context of a Housing First (HF) randomized trial and approach that also offered intensive case management (ICM), including ethnoracial-specific ICM services for racialized individuals as an option, alongside ACT (59). No information was reported regarding the healthcare professionals' roles; however, case managers were noted to have an integral role in the care and coordination of clients (57, 59). Other members of the rehabilitation team included psychiatrists and peer support workers or peer support specialists (57, 59).

All four articles integrated TBI care in their programs or interventions through TBI screening (46, 51, 57, 59) and/or tailoring interventions to accommodate for TBI impairments that are often cognitive in nature (46, 51, 57). Face-to-face interviews were conducted (59) or the OSU-TBI-ID (46, 51, 57) were administered by trained healthcare professionals, including case managers from varying disciplinary backgrounds, OTs, and social workers, to screen for TBI. In two studies, screening for TBI was followed by functional assessments through standardized assessments or observation, to understand how cognitive limitations interfere with an individual's ability to engage in activities (46, 51). All four articles highlighted the importance of TBI screening and/or identifying TBI-related impairments that impact daily functioning to develop strategies that accommodate such impairments (46, 51, 57, 59).

Three of the four articles described strategies for tailoring interventions to accommodate TBI-related impairments, including strategies related to the treatment session (51, 57) or the treatment environment (46). Intervention-related strategies included setting short-term, specific, measurable, achievable, realistic, and time-limited (SMART) goals (51), having shorter sessions over a longer period of time and/or booster sessions, providing patients with frequent reminders, written treatment plans and short verbal summaries using lay language, short schedules, one-on-one communication in a quiet environment, and establishing a routine (57). Environmental-related strategies included modifications to tailor the physical environment specifically for patients with TBI and involved implementing organizational aids such as calendars, program schedules, and white boards with reminders (46). One study that focused on provider training categorized such strategies into external or internal strategies (51). Examples of external strategies included structuring intervention sessions and health professionals supporting patients in applying strategies discussed during the session. Examples of internal strategies included stress management and self-soothing strategies to accommodate for deficits related to attention, self-awareness, and self-management (51).

#### Rehabilitation interventions for individuals experiencing homelessness without specific inclusion criteria for TBI (N = 11)

All eight articles and three gray literature reports described rehabilitation within a multidisciplinary care context. They all focused on individuals experiencing homelessness (45, 49, 50, 52, 54–56, 58, 60–62). TBI was not an inclusion criterion for these studies but up to 80% reported their participants had a history of TBI. However, no information regarding screening was provided. Seven of these articles utilized a HF approach (45, 55, 56, 58, 60–62), a recovery-oriented approach that involves immediate provision of housing without pre-conditions followed by necessary services and supports (55, 59). These studies employed either ACT or ICM interventions, which involved multidisciplinary teams of nurses, psychiatrists, social workers, rehabilitation workers, recreation therapists, nutritionists, substance abuse workers, and peer support workers, or case managers that facilitated access to services, to deliver community-based supports that were tailored toward individual need and level of disability. One article utilized harm-reduction and peer-support approaches alongside HF (45). Two studies focused on individuals with SUD who were experiencing homelessness. The first study utilized an integrated treatment approach for individuals with SUD and severe mental illness. Treatment was administered by senior clinicians and involved case management, an evidence-based SUD group intervention, contingency management to reduce substance use, and relapse prevention interventions (49). The second study described a mobile health outreach program for individuals experiencing homelessness in response to the opioid overdose crisis (54). The program involved a mobile unit consisting of addiction medicine clinicians, public health advocates, and harm reduction specialists who delivered primary care, addiction treatment, and harm reduction services to four locations on the same day at the same time each week. The remaining studies in this category described an occupational therapy intervention at an integrated healthcare site or Federally Qualified Health Centers (FQHC) that provided comprehensive services for individuals experiencing homelessness (50, 52). Occupational therapy services included an evaluation, individualized intervention focusing on client-identified goals and occupational performance, and consultation (e.g., discussion and treatment planning with another provider and evaluation of environments). Other services that were part of the FQHC were described briefly as encompassing medical care, chronic disease management, counseling for mental health and addiction, case management, and supportive housing, and included physicians, nurses, case managers, and social workers.

Interventions in this category did not detail strategies for tailoring interventions for TBI-related impairments. It is worth noting, however, that the studies on the occupational therapy intervention referred to TBI as a complex medical condition and specifically reported that TBI, a history of head trauma, or medical conditions that affected cognition constituted most referrals for an occupational therapy evaluation (50, 52). Providers in the FQHC also noted the value of occupational therapy in providing an in-depth understanding of cognitive challenges and their impact on an individual's daily life activities and subsequently determining necessary supports (50).

#### Rehabilitation service use without specific inclusion criteria for experiences of homelessness or TBI (N = 3)

A key distinction between studies in this category (47, 48, 53) and those identified in the first two categories is that experiences of homelessness and/or TBI were not inclusion criteria for participating in the research study. These specialized programs included a vocational rehabilitation program (48), an emergency services outreach program (53), and an occupational therapy intervention for women experiencing homelessness and/or domestic violence (47). The vocational rehabilitation program aimed to transition and integrate individuals from five different settings into community living; one of the settings was homeless shelters, of whom 1.1% of individuals experienced TBI (48). The emergency services outreach program utilized outreach and ICM services to engage frequent users of emergency services, of whom 88% were individuals who are homeless and have chronic mental illness and SUD; and 16.6% had a history of TBI. Services were provided exclusively by a case manager who directly interacted with frequent users of emergency services to provide tailored treatment plans and support in accessing relevant resources (53). The occupational therapy intervention for women experiencing homelessness and/or domestic violence was designed to address possible cognitive impairment sustained from domestic violence, of which 50% of women were experiencing homelessness, 19% self-reported sustaining a TBI from domestic violence, and 62% had some form of cognitive impairment documented in their chart (47). This intervention involved OTs addressing a broad range of participant-identified needs including safety planning, drug and alcohol awareness, safe sex practices, assertiveness training, anger and stress management, vocational and educational skill training, money management, housing support, leisure exploration, and health maintenance in a not-for-profit community organization. This article was the only one of the three that reported on strategies to address possible TBI or cognitive impairments and incorporated key attributes of TBI-related rehabilitation, such as the length of time needed for change and the non-linearity of the recovery process (47). None of the articles conducted or reported on TBI screening.

## Discussion

This scoping review explored the extent to which rehabilitation, including the types of rehabilitation interventions, is available to, or used by, individuals experiencing homelessness and TBI. A systematic search identified four articles focused specifically on rehabilitation for individuals experiencing homelessness and TBI (46, 51, 57, 59), 11 articles on individuals experiencing homelessness (45, 49, 50, 52, 54–56, 58, 60–62), and the remainder on general use of rehabilitation services (47, 48, 53). A broad range of multidisciplinary and specialized rehabilitation programs and/or interventions were provided by OTs, social workers, case managers, psychiatrists, registered nurses, physicians, addiction medicine and primary care clinicians, harm reduction specialists, community health workers, public health advocates, peer support specialists, and/or peer-support workers. This scoping review demonstrates that rehabilitation programs or interventions for individuals experiencing homelessness and TBI already exist. Furthermore, rehabilitation focused on individuals experiencing homelessness are already serving individuals with TBI. However, interventions described by these articles did not consider TBI in the program or intervention despite the high proportion of participants with TBI (up to 80%) (45, 47–50, 52–56, 58, 60–62). Only five articles integrated TBI in their rehabilitation programs or interventions by explicitly screening for TBI and/or including intervention- or environment-related accommodations for TBI-related impairments (46, 47, 51, 57, 59). Below, we discuss key findings in relation to (a) opportunities to adapt existing rehabilitation for individuals who experience homelessness and TBI, (b) considerations for education and training on TBI, and (c) recommendations for future research.

### Opportunities to adapt existing rehabilitation for individuals who experience homelessness and TBI

Findings from this scoping review highlight opportunities to adapt existing rehabilitation programs and services through (a) screening for TBI, (b) conducting cognitive and functional assessments, and (c) tailoring interventions with multidisciplinary teams. Screening for TBI was highlighted as a critical first step in identifying clients with TBI so that interventions can be tailored to address TBI-related impairments (46, 51, 57). This finding on the importance of screening is not unique to this review, as prior research has noted that screening for TBI facilitates the identification of and access to appropriate services and supports to individuals who need them (63). Clinical interviews and self-reports of TBI identified through validated screening tools are considered the gold standard for identifying lifetime history of exposure to TBI (64), and are also found to be beneficial in identifying a history of TBI in community samples and among underserved populations (63, 65). However, a key finding from this review is the potential benefit of conducting cognitive and functional assessments following screening for TBI. Doing so provided an opportunity to not only identify cognitive limitations associated with TBI but to also gain an in-depth understanding of how such limitations impact an individual's functional abilities as well as priority areas for treatment (46, 47, 50–52). Specifically, these assessments allowed for targeted intervention- (46, 47, 51, 57) and environment-related accommodations (46) that account for TBI-related challenges such as difficulties with recall, organization, problem solving, and frustration tolerance (46) and the possible implications of these limitations on treatment (e.g., longer duration of treatment and smaller gains and non-linear trajectory of recovery) (47). The value of accommodations for TBI has been recognized outside of articles included in this review (66, 67). Importantly, it holds the potential to better support individuals with TBI to maintain stable housing, as the provision of housing without addressing TBI-related challenges may put these individuals at continued risk of experiencing homelessness (67).

Equally important is the beneficial role of a multidisciplinary team in delivering the above-mentioned rehabilitation adaptations. Notably, almost all the articles that described adaptations were provided within a multidisciplinary context consisting of OTs, social workers, case managers, psychiatrists, registered nurses, clinicians, physicians, rehabilitation workers, recreation therapists, nutritionists, substance abuse workers, community health workers, peer support specialists, and/or peer-support workers (46, 47, 51, 57, 59). We acknowledge that we are unable to comment on the effectiveness of the multidisciplinary rehabilitation programs or interventions identified in this review due to potential biases identified during the quality appraisal, including but not limited to selection, reporting, and publication bias. However, multidisciplinary rehabilitation has been demonstrated to result in improved outcomes post-TBI and cost-related savings for the individuals and society (68–71). As such, utilizing multidisciplinary teams to screen for TBI, conduct functional and cognitive assessments, and adapt programs and/or interventions to accommodate TBI-related impairments should be explored and its impact on outcomes assessed. Research into barriers and facilitators for multidisciplinary rehabilitation, particularly those offered in the community setting, for individuals experiencing homelessness and TBI is also encouraged to inform opportunities to utilize multidisciplinary rehabilitation in this setting. In particular, the introduction of multidisciplinary teams that can provide accommodations for TBI may be beneficial in supporting individuals with TBI who are already receiving rehabilitation for homelessness.

### Considerations for education and training on TBI

Education and training for healthcare professionals working with individuals experiencing homelessness and TBI should be further examined and include structured education and training sessions, collaboration with a multidisciplinary team, and co-development of educational materials with service users. First, the need for education and training in working with individuals with a history of TBI has been explicitly noted in three articles in this review, with healthcare providers highlighting the value of receiving structured training on screening and functional assessments for TBI, as well as training related to the development of concrete strategies to address the needs of those with TBI (46, 51, 57). This is because individuals with TBI were often viewed as complex and referred to other healthcare professionals such as OTs (50, 52) or discharged early due to aggressive behaviors (57). Clinicians were not aware of the impact of TBI history on rehabilitation (57) and noted the value of having another healthcare professional such as an OT providing an in-depth assessment of cognitive and functional limitations associated with TBI (46, 50). The view that TBI is a complex condition, and the need for, and importance of, formal TBI education and training has been identified in research outside of those included in this review. For example, a qualitative study sought the perspectives of housing services providers in Canada and identified attitudes around TBI that affected service delivery (72). Specifically, providers referred to TBI as an unknown and reported lacking TBI knowledge and expertise or needing to go “above and beyond” their role to support individuals with TBI in finding and maintaining housing (72).

Multidisciplinary teams may also facilitate opportunities for education on TBI. Alongside the call for formal education and training, housing service providers have also reported benefitting from partnerships and collaborations with healthcare professionals who had knowledge and expertise in working with individuals with TBI (72). This is particularly important given the intersecting challenges faced by individuals experiencing homelessness and TBI reported in articles included in this scoping review, including but not limited to criminal justice involvement (45, 49, 56, 58, 60, 61) domestic violence (47), and comorbid mental health and substance use (MHSU) challenges (45, 47–61). In fact, a systematic review on integrated care for individuals with TBI and MHSU found that multidisciplinary teams, or informal meetings and discussions between different healthcare disciplines, may offer opportunities for education. This was acknowledged to be important, as the lack of experience with TBI and MHSU was a noted barrier to diagnosis, contributing to delayed treatment (73). Thus, opportunities for formal and informal education and training on TBI, screening, assessments, and adapted interventions should be explored.

Co-developing education materials on TBI with service users and service providers should be considered, including screening, assessments, and adapted interventions. For example, research to explore and develop screening protocols for TBI should be conducted. While screening tools for TBI exist (e.g., OSU-TBI-ID that were also used in articles identified in this review), there is limited research on the feasibility and validity of using these screening tools for individuals experiencing homelessness (63, 74–76). Importantly, while screening and/or a diagnosis of a TBI may provide opportunities to adapt interventions to accommodate for TBI-related impairments, it is acknowledged that a TBI diagnosis may also be a barrier to other treatments, particularly those for MHSU, as interventions may have exclusion criteria based on a history of TBI or cognitive impairment (77). This finding is particularly important for individuals experiencing homelessness, given the prevalence of comorbid MHSU (45, 47–61) and intersecting experiences and challenges (45, 49, 56, 58, 60, 61). As such, collaborative research that engages individuals with lived experience of TBI and homelessness on the advantages and disadvantages of screening for TBI should be conducted to inform considerations when implementing screening and to mitigate unintended consequences of TBI screening.

### Opportunities for future research

Research that considers the rehabilitation needs of diverse individuals experiencing homelessness and TBI is urgently needed. None of the articles identified in this review utilized an intersectional lens or considered intersecting identities in their rehabilitation programs/interventions; only a few articles reported on a HF approach that utilized ethnoracial ICM, anti-oppression approaches (58, 59), and HF programs that developed Aboriginal activities (e.g., healing circles and annual pow-wow) and support activities for gender diverse individuals (62). We acknowledge that the lack of articles may be reflective of our search strategy, as we did not explicitly include search terms related to intersecting identities. However, the intersectionality of sex, gender, race, ethnicity, disability, and other social identities leads to unique health experiences that cannot be addressed by looking at a single facet of identity. It is also noteworthy that, of the articles that reported age eligibility for their programs or interventions, none included youths in their eligibility criteria. As such, rehabilitation programs and interventions that consider diverse experiences of individuals experiencing homelessness and TBI across the age continuum must be available to address health equity and universal access to quality healthcare (78). Research that examines equity considerations in clinical practice guidelines (CPG) for TBI and homelessness are also encouraged. This is particularly important because CPGs are “statements that include recommendations intended to optimize patient care” (79) and used to reduce inappropriate variations in practice and enhance safety and quality of healthcare (79). However, it has been highlighted that most studies included in CPGs for TBI are population-based and do not consider the diversity of patients with TBI and, as a result, promotes a one-size-fits all approach to care (80). As such, healthcare providers using CPGs should be aware that existing recommendations may not take into account unique healthcare needs and challenges experienced by individuals experiencing homelessness and TBI. Systematic reviews of existing CPGs for TBI and homelessness to assess the extent to which evidence about homelessness and TBI is integrated in these CPGs hold the potential to provide an evidence-based foundation to advance equity considerations in CPGs.

### Strengths and limitations

We acknowledge the following limitations. First, only published peer-reviewed articles or gray literature were identified; this may result in the omission of rehabilitation programs or interventions that were never formally reported or presented. However, we aimed to minimize publication bias by consulting with our PAC and searching for gray literature to capture non-peer-reviewed reports that may describe services offered by community-based organizations serving individuals experiencing homelessness and/or TBI. We also aimed to minimize language bias by including non-English language peer-reviewed articles in our search. Second, this scoping review only included articles that focused on individuals who were experiencing homelessness at the time of the research study; this excludes individuals at risk of homelessness, defined as individuals who are “not homelessness, but current economic and/or housing situation is precarious or does not meet public health and safety standards” (38). We acknowledge that homelessness is a fluid experience and that homeless and vulnerably housed individuals may experience similar unmet healthcare needs. However, research articles focused on individuals experiencing homelessness may describe a different rehabilitation experience than articles that focus on individuals at risk of homelessness. Thus, we believe this exclusion was appropriate and aligns with the overall scope of this review. Future reviews on rehabilitation focused on specific populations at risk of homelessness, as well as individuals with lived experience of homelessness and are now in permanent housing, are encouraged. Third, we acknowledge that the inclusion of a quality appraisal is not consistent with scoping review methodologies outlined in this review (34, 35); no articles were eliminated as a result of the quality appraisals and results were used to inform the discussion of our findings. We recognize potential biases in the articles included in this scoping review and acknowledge we are unable to evaluate the impact of the rehabilitation programs or interventions. Finally, while we did not restrict our search by geography, only studies conducted in the United States or Canada were identified and/or met inclusion criteria to be included in this review; thus, findings may not be generalizable to other countries or health systems due to differences in culture, resources, and social behaviors.

A major strength of our scoping review is that it was guided by scoping review methodology frameworks to address methodological rigor, which has been highlighted as a limitation of existing scoping reviews on rehabilitation (81). Furthermore, as part of Stage 6, feedback from stakeholders of our scoping review were received and integrated in the interpretation of findings from this review. The charting of the data also explicitly identified intersecting sex, gender, social identities and vulnerabilities, including intersections with the criminal justice system, which is common among individuals experiencing homelessness and TBI (82). Finally, our search strategy was purposely broad, to identify articles that explore the concepts of homelessness and rehabilitation, or homelessness and TBI. In addition, the title and abstract screen included articles that explored the broader brain-injured population without specific mention of TBI. The inclusion of these articles at the title and abstract screen reduced the risk of omitting relevant articles.

## Conclusion

To the best of our knowledge, this is the first scoping review that explored the extent to which rehabilitation, including the types of rehabilitation interventions, are available to or used by, individuals experiencing homelessness and TBI. Rehabilitation programs or interventions for this population already exist, and those that are focused on individuals experiencing homelessness are already serving individuals with TBI. Opportunities to introduce multidisciplinary teams that screen for TBI, assess functioning and cognition of individuals, and tailor programs and/or interventions to accommodate TBI-related impairments should be explored to maximize the benefit of rehabilitation for this population. In particular, the introduction of accommodations for TBI holds the potential to better support individuals with TBI who are already receiving rehabilitation for homelessness. Similarly, opportunities for formal and informal education and training on TBI, screening, assessments, and treatments should be considered. These include research with service users and providers to co-develop education materials to better equip service providers with appropriate tools and knowledge to support individuals experiencing homelessness and TBI. Finally, research engaging individuals with lived experience of homelessness and TBI are urgently needed to inform considerations when developing and implementing TBI screening protocols and to better understand the rehabilitation needs of diverse individuals. An examination of equity considerations in existing CPGs for TBI is also encouraged to provide an evidence-based foundation to advance equity considerations in rehabilitation care for individuals experiencing homelessness and TBI.

## Author contributions

VC and AC conceptualized this scoping review. VC, MJE, and JB developed the search strategy. VC and MJE formulated the design. VC, MJE, RB, and RS screened the articles, charted the data, and/or completed the quality appraisal of the included articles. VC, MJE, and RB completed the analyses for this review and drafted the manuscript. All authors critically reviewed the manuscript and approved the final manuscript.

## Funding

This study was supported by the Canada Research Chairs Program (Grant # N/A) and the Ontario Ministry of Health and Long-Term Care, Grant #725A.

## Conflict of interest

The authors declare that the research was conducted in the absence of any commercial or financial relationships that could be construed as a potential conflict of interest.

## Publisher's note

All claims expressed in this article are solely those of the authors and do not necessarily represent those of their affiliated organizations, or those of the publisher, the editors and the reviewers. Any product that may be evaluated in this article, or claim that may be made by its manufacturer, is not guaranteed or endorsed by the publisher.

## Author disclaimer

The views expressed in this publication are those of the authors and do not necessarily reflect those of the Ministry of Health and Long-Term Care.

## Supplementary material

Supplementary File 1 Search strategy.

Supplementary File 2 Charting table.

Supplementary File 3 Quality appraisal.
