# Supplementary material for: Rehabilitation among individuals experiencing homelessness and traumatic brain injury: A scoping review
Source: Front Med (Lausanne). 2022 Nov 11;9:916602. doi: 10.3389/fmed.2022.916602 (PMC9692012; doi:10.3389/fmed.2022.916602)
Supplement: Supplementary File 1 — Search strategy. [file Data_Sheet_1.pdf]

Supplementary File 1: Search Strategy

Database: MEDLINE(R) ALL <1946 to March 22, 2022>
Platform: Ovid
Last searched: March 23 2022

1 exp Homeless persons/ (10293)
2 homeless*.tw,kf. (12524)
3 Roofless*.tw,kf. (19)
4 (Marginal* adj3 hous*).tw,kf. (222)
5 (precarious* adj3 hous*).tw,kf. (116)
6 (unstabl* adj3 hous*).tw,kf. (644)
7 (instab* adj3 hous*).tw,kf. (542)
8 (interim* adj3 hous*).tw,kf. (9)
9 (temporary adj3 (liv* or hous*)).tw,kf. (702)
10 ((liv* or sleep* or stay or emergenc*) adj3 shelter??).tw,kf. (848)
11 houseless*.tw,kf. (15)
12 unsheltered.tw,kf. (122)
13 rough sleeper?.tw,kf. (38)
14 rough sleeping.tw,kf. (25)
15 provisionally accommodat*.tw,kf. (0)
16 or/1-15 (16989)
17 "Physical and Rehabilitation Medicine"/ (3478)
18 exp rehabilitation/ (335825)
19 rehab*.tw,kf,jw. (256099)
20 telerehab*.tw,kf,jw. (1446)
21 neurorehab*.tw,kf,jw. (8270)
22 rh.fs. (206135)
23 (physiatrist? or physiatry).tw,kf. (1599)
24 Rehabilitation Centers/ (8518)
25 occupational therapy/ (14170)
26 (occupational adj therap*).tw,kf,jw. (16671)
27 physical therapy specialty/ (2955)
28 (physical adj therap*).tw,kf,jw. (29473)
29 physiotherap*.tw,kf,jw. (37130)
30 physio-therapist*.tw,kf,jw. (11)
31 Speech-Language Pathology/ (3477)
32 (speech adj2 (therap* or patholog*)).tw,kf,jw. (11111)
33 Neuropsychology/ (2529)
34 Neuropsycholog*.tw,kf,jw. (81766)
35 Nutritionists/ (1572)
36 (Nutritionist? or Dietician?).tw,kf,jw. (5360)
37 (therap* adj recreation*).tw,kf,jw. (211)
38 child life specialist?.tw,kf. (177)
39 play therapy/ (1183)
40 (play adj therap*).tw,kf. (503)
41 Respite Care/ (1076)
42 respite.tw,kf. (2020)
43 or/17-42 (742067)

44 exp Brain Injuries/ (76910)
45 exp Brain Injuries, Traumatic/ (20515)
46 exp Brain Concussion/ (10977)
47 Craniocerebral Trauma/ (23053)
48 tbi*2.tw,kf. (31547)
49 mtbi*2.tw,kf. (3596)
50 concuss*.tw,kf. (11686)
51 postconcuss*.tw,kf. (1569)
52 ((head* or brain* or cerebr* or crani* or skull* or intracran*) adj2 (injur* or trauma* or damag* or wound* or swell* or oedema* or edema* or fracture* or contusion* or pressur*)).tw,kf,jw. (192434)
53 ((brain* or cerebr* or intracerebr* or crani* or intracran* or head* or subdural* or epidural* or extradural*) adj (haematoma* or hematoma* or hemorrhag* or haemorrhag* or bleed*)).tw,kf. (59033)
54 exp cognition disorders/ (107912)
55 ((cogniti* or neurocogniti*) adj2 (impair* or dysfunction* or disorder* or declin*)).tw,kf. (134708)
56 or/44-55 (450668)
57 16 and 43 (1618)
58 16 and 56 (251)
59 57 or 58 (1801)
60 59 not (exp animals/ not humans.sh.) (1796)

****

Database: Cochrane Central Register of Controlled Trials <January 2022>
Platform: Ovid
Last searched: March 23 2022

1 exp Homeless persons/ (398)
2 homeless*.tw,kw. (1083)
3 Roofless*.tw,kw. (0)
4 (Marginal* adj3 hous*).tw,kw. (28)
5 (precarious* adj3 hous*).tw,kw. (17)
6 (unstabl* adj3 hous*).tw,kw. (59)
7 (instab* adj3 hous*).tw,kw. (43)
8 (interim* adj3 hous*).tw,kw. (8)
9 (temporary adj3 (liv* or hous*)).tw,kw. (43)
10 ((liv* or sleep* or stay or emergenc*) adj3 shelter??).tw,kw. (92)
11 houseless*.tw,kw. (0)
12 unsheltered.tw,kw. (4)
13 rough sleeper?.tw,kw. (1)
14 rough sleeping.tw,kw. (0)
15 provisionally accommodat*.tw,kw. (1)
16 or/1-15 (1297)
17 "Physical and Rehabilitation Medicine"/ (20)
18 exp rehabilitation/ (27148)
19 rehab*.tw,kw,jw. (48882)
20 telerehab*.tw,kw,jw. (737)
21 neurorehab*.tw,kw,jw. (1410)
22 rh.fs. (18916)
23 (physiatrist? or physiatry).tw,kw. (206)

24 Rehabilitation Centers/ (321)
25 occupational therapy/ (813)
26 (occupational adj therap*).tw,kw,jw. (3444)
27 physical therapy specialty/ (119)
28 (physical adj therap*).tw,kw,jw. (9057)
29 physiotherap*.tw,kw,jw. (19078)
30 physio-therapist*.tw,kw,jw. (8)
31 Speech-Language Pathology/ (80)
32 (speech adj2 (therap* or patholog*)).tw,kw,jw. (1856)
33 Neuropsychology/ (22)
34 Neuropsycholog*.tw,kw,jw. (9955)
35 Nutritionists/ (53)
36 (Nutritionist? or Dietician?).tw,kw,jw. (1634)
37 (therap* adj recreation*).tw,kw,jw. (32)
38 child life specialist?.tw,kw. (43)
39 play therapy/ (74)
40 (play adj therap*).tw,kw. (194)
41 Respite Care/ (15)
42 respite.tw,kw. (175)
43 or/17-42 (106855)
44 exp Brain Injuries/ (2359)
45 exp Brain Injuries/ (2359)
46 exp Brain Concussion/ (442)
47 Craniocerebral Trauma/ (338)
48 tbi*2.tw,kw. (3602)
49 mtbi*2.tw,kw. (429)
50 concuss*.tw,kw. (882)
51 postconcuss*.tw,kw. (289)
52 ((head* or brain* or cerebr* or crani* or skull* or intracran*) adj2 (injur* or trauma* or damag* or wound* or swell* or oedema* or edema* or fracture* or contusion* or pressur*)).tw,kw,jw. (14861)
53 ((brain* or cerebr* or intracerebr* or crani* or intracran* or head* or subdural* or epidural* or extradural*) adj (haematoma* or hematoma* or hemorrhag* or haemorrhag* or bleed*)).tw,kw. (8662)
54 exp cognition disorders/ (3869)
55 ((cogniti* or neurocogniti*) adj2 (impair* or dysfunction* or disorder* or declin*)).tw,kw. (19462)
56 or/44-55 (44653)
57 16 and 43 (186)
58 16 and 56 (26)
59 57 or 58 (206)
60 "https://clinicaltrials.gov*".so. (214653)
61 "https://trialsearch.who.int*".so. (166490)
62 59 not (60 or 61) (177)

****

Database: Embase Classic+Embase <1947 to 2022 March 22>
Platform: Ovid
Last searched: March 23 2022

1 exp Homeless person/ (3162)

2 homeless*.tw,kw. (15454)
3 Roofless*.tw,kw. (23)
4 (Marginal* adj3 hous*).tw,kw. (281)
5 (precarious* adj3 hous*).tw,kw. (140)
6 (unstabl* adj3 hous*).tw,kw. (938)
7 (instab* adj3 hous*).tw,kw. (659)
8 (interim* adj3 hous*).tw,kw. (23)
9 (temporary adj3 (liv* or hous*)).tw,kw. (927)
10 ((liv* or sleep* or stay or emergenc*) adj3 shelter??).tw,kw. (1018)
11 houseless*.tw,kw. (18)
12 unsheltered.tw,kw. (160)
13 rough sleeper?.tw,kw. (45)
14 rough sleeping.tw,kw. (34)
15 provisionally accommodat*.tw,kw. (0)
16 or/1-15 (18751)
17 rehabilitation medicine/ or physical medicine/ (18936)
18 exp rehabilitation/ (464374)
19 rehab*.tw,kw,jx. (391982)
20 telerehab*.tw,kw,jx. (1623)
21 neurorehab*.tw,kw,jx. (12318)
22 rh.fs. (168274)
23 (physiatrist? or physiatry).tw,kw. (2962)
24 Rehabilitation Center/ (18423)
25 occupational therapy/ (27150)
26 (occupational adj therap*).tw,kw,jx. (31937)
27 exp physiotherapy/ (107099)
28 (physical adj therap*).tw,kw,jx. (53790)
29 physiotherap*.tw,kw,jx. (69646)
30 physio-therapist*.tw,kw,jx. (34)
31 "speech and language rehabilitation"/ (898)
32 (speech adj2 (therap* or patholog*)).tw,kw,jx. (17086)
33 Neuropsychology/ (19991)
34 Neuropsycholog*.tw,kw,jx. (114845)
35 Dietitian/ (14791)
36 (Nutritionist? or Dietician?).tw,kw,jx. (10536)
37 (therap* adj recreation*).tw,kw,jx. (371)
38 child life specialist?.tw,kw. (430)
39 play therapy/ (1984)
40 (play adj therap*).tw,kw. (791)
41 Respite Care/ (1223)
42 respite.tw,kw. (2744)
43 or/17-42 (1030390)
44 exp Brain Injury/ (209238)
45 exp traumatic brain injury/ (59298)
46 Brain concussion/ or postconcussion syndrome/ (10044)
47 head injury/ (56852)
48 tbi*2.tw,kw. (51486)
49 mtbi*2.tw,kw. (5595)
50 concuss*.tw,kw. (16367)
51 postconcuss*.tw,kw. (2052)

52 ((head* or brain* or cerebr* or crani* or skull* or intracran*) adj2 (injur* or trauma* or damag* or wound* or swell* or oedema* or edema* or fracture* or contusion* or pressur*)).tw,kw,jx. (265699)
53 ((brain* or cerebr* or intracerebr* or crani* or intracran* or head* or subdural* or epidural* or extradural*) adj (haematoma* or hematoma* or hemorrhag* or haemorrhag* or bleed*)).tw,kw. (89204)
54 exp cognitive defect/ (559940)
55 ((cogniti* or neurocogniti*) adj2 (impair* or dysfunction* or disorder* or declin*)).tw,kw. (200691)
56 or/44-55 (1030242)
57 16 and 43 (1945)
58 16 and 56 (528)
59 57 or 58 (2345)
60 59 not medline.cr. (1753)
61 60 not (((rat or rats or mouse or mice or swine or porcine or murine or sheep or lambs or pigs or piglets or rabbit or rabbits or cat or cats or dog or dogs or cattle or bovine or monkey or monkeys or trout or marmoset$1).ti. and animal experiment/) or (Animal experiment/ not (human experiment/ or human/))) (1745)

****

Database: APA PsycInfo <1806 to March Week 2 2022>
Platform: Ovid
Last searched: March 23 2022

1 exp Homeless/ or shelters/ (9244)
2 homeless*.ti,ab. (11859)
3 Roofless*.ti,ab. (14)
4 (Marginal* adj3 hous*).ti,ab. (106)
5 (precarious* adj3 hous*).ti,ab. (71)
6 (unstabl* adj3 hous*).ti,ab. (399)
7 (instab* adj3 hous*).ti,ab. (394)
8 (interim* adj3 hous*).ti,ab. (9)
9 (temporary adj3 (liv* or hous*)).ti,ab. (228)
10 ((liv* or sleep* or stay or emergenc*) adj3 shelter??).ti,ab. (866)
11 houseless*.ti,ab. (15)
12 unsheltered.ti,ab. (72)
13 rough sleeper?.ti,ab. (22)
14 rough sleeping.ti,ab. (28)
15 provisionally accommodat*.ti,ab. (0)
16 or/1-15 (14350)
17 exp rehabilitation/ (52371)
18 rehab*.ti,ab,jx. (82286)
19 telerehab*.ti,ab,jx. (192)
20 neurorehab*.ti,ab,jx. (4793)
21 (physiatrist? or physiatry).ti,ab. (146)
22 exp Rehabilitation Centers/ (1165)
23 occupational therapy/ (6742)
24 (occupational adj therap*).ti,ab,jx. (14624)
25 Physical therapy/ (3237)
26 (physical adj therap*).ti,ab,jx. (3917)
27 physiotherap*.ti,ab,jx. (3514)
28 physio-therapist*.ti,ab,jx. (0)
29 Speech Therapists/ (1419)

30 (speech adj2 (therap* or patholog*)).ti,ab,jx. (10072)
31 Neuropsychology/ (20750)
32 Neuropsycholog*.ti,ab,jx. (82252)
33 (Nutritionist? or Dietician?).ti,ab,jx. (780)
34 (therap* adj recreation*).ti,ab,jx. (588)
35 child life specialist?.ti,ab. (118)
36 play therapy/ (3975)
37 (play adj therap*).ti,ab. (3388)
38 Respite Care/ (476)
39 respite.ti,ab. (1746)
40 or/17-39 (210998)
41 exp Brain Injuries/ (22365)
42 exp traumatic brain injury/ (21593)
43 Brain concussion/ (2799)
44 head injuries/ (4650)
45 tbi*2.ti,ab. (12157)
46 mtbi*2.ti,ab. (2189)
47 concuss*.ti,ab. (3738)
48 postconcuss*.ti,ab. (878)
49 ((head* or brain* or cerebr* or crani* or skull* or intracran*) adj2 (injur* or trauma* or damag* or wound* or swell* or oedema* or edema* or fracture* or contusion* or pressur*)).ti,ab,jx. (51578)
50 ((brain* or cerebr* or intracerebr* or crani* or intracran* or head* or subdural* or epidural* or extradural*) adj (haematoma* or hematoma* or hemorrhag* or haemorrhag* or bleed*)).ti,ab. (3598)
51 Cognitive Impairment/ or Mild Cognitive Impairment/ (42338)
52 ((cogniti* or neurocogniti*) adj2 (impair* or dysfunction* or disorder* or declin*)).ti,ab. (70345)
53 or/41-52 (135225)
54 16 and 40 (872)
55 16 and 53 (193)
56 54 or 55 (1003)
57 limit 56 to ("column/opinion" or dissertation or editorial) (101)
58 56 not 57 (902)
59 limit 58 to animal (9)
60 limit 58 to human (872)
61 58 not (59 not 60) (897)

****

Database: CINAHL Complete (Thursday, March 24, 2022)
Search Strategy:

#	Query	Limiters/Expanders	Last Run Via	Results
S1	(MH "Homelessness")	Expanders - Apply equivalent subjects<br>Search modes - Boolean/Phrase	Interface - EBSCOhost Research Databases<br>Search Screen - Advanced Search<br>Database - CINAHL Complete	4,234
S2	(MH "Homeless Persons")	Expanders - Apply equivalent subjects<br>Search modes - Boolean/Phrase	Interface - EBSCOhost Research Databases<br>Search Screen - Advanced	5,952

			Search<br>Database - CINAHL Complete	
S3	TI homeless* OR AB homeless*	Expanders - Apply equivalent subjects<br>Search modes - Boolean/Phrase	Interface - EBSCOhost Research Databases<br>Search Screen - Advanced Search<br>Database - CINAHL Complete	9,103
S4	TI Roofless* OR AB Roofless*	Expanders - Apply equivalent subjects<br>Search modes - Boolean/Phrase	Interface - EBSCOhost Research Databases<br>Search Screen - Advanced Search<br>Database - CINAHL Complete	7
S5	TI (Marginal* n3 hous*) OR AB (Marginal* n3 hous*)	Expanders - Apply equivalent subjects<br>Search modes - Boolean/Phrase	Interface - EBSCOhost Research Databases<br>Search Screen - Advanced Search<br>Database - CINAHL Complete	134
S6	TI (precarious* n3 hous*) OR AB (precarious* n3 hous*)	Expanders - Apply equivalent subjects<br>Search modes - Boolean/Phrase	Interface - EBSCOhost Research Databases<br>Search Screen - Advanced Search<br>Database - CINAHL Complete	64
S7	TI (unstabl* n3 hous*) OR AB (unstabl* n3 hous*)	Expanders - Apply equivalent subjects<br>Search modes - Boolean/Phrase	Interface - EBSCOhost Research Databases<br>Search Screen - Advanced Search<br>Database - CINAHL Complete	380
S8	TI (instab* n3 hous*) OR AB (instab* n3 hous*)	Expanders - Apply equivalent subjects<br>Search modes - Boolean/Phrase	Interface - EBSCOhost Research Databases<br>Search Screen - Advanced Search<br>Database - CINAHL Complete	327
S9	TI (interim* n3 hous*) OR AB (interim* n3 hous*)	Expanders - Apply equivalent subjects<br>Search modes - Boolean/Phrase	Interface - EBSCOhost Research Databases<br>Search Screen - Advanced Search<br>Database - CINAHL Complete	8
S10	TI ((temporary n3 (liv* or hous*))) OR AB ((temporary n3 (liv* or hous*)))	Expanders - Apply equivalent subjects<br>Search modes - Boolean/Phrase	Interface - EBSCOhost Research Databases<br>Search Screen - Advanced Search<br>Database - CINAHL Complete	212
S11	TI (((liv* or sleep* or stay or emergenc*) n3 shelter*)) OR AB (((liv* or sleep* or stay or emergenc*) n3 shelter*))	Expanders - Apply equivalent subjects<br>Search modes - Boolean/Phrase	Interface - EBSCOhost Research Databases<br>Search Screen - Advanced Search<br>Database - CINAHL Complete	521

S12	TI houseless* OR AB houseless*	Expanders - Apply equivalent subjects<br>Search modes - Boolean/Phrase	Interface - EBSCOhost Research Databases<br>Search Screen - Advanced Search<br>Database - CINAHL Complete	10
S13	TI unsheltered OR AB unsheltered	Expanders - Apply equivalent subjects<br>Search modes - Boolean/Phrase	Interface - EBSCOhost Research Databases<br>Search Screen - Advanced Search<br>Database - CINAHL Complete	46
S14	TI rough sleeper* OR AB rough sleeper*	Expanders - Apply equivalent subjects<br>Search modes - Boolean/Phrase	Interface - EBSCOhost Research Databases<br>Search Screen - Advanced Search<br>Database - CINAHL Complete	51
S15	TI rough sleeping OR AB rough sleeping	Expanders - Apply equivalent subjects<br>Search modes - Boolean/Phrase	Interface - EBSCOhost Research Databases<br>Search Screen - Advanced Search<br>Database - CINAHL Complete	27
S16	TI provisionally accommodat* OR AB provisionally accommodat*	Expanders - Apply equivalent subjects<br>Search modes - Boolean/Phrase	Interface - EBSCOhost Research Databases<br>Search Screen - Advanced Search<br>Database - CINAHL Complete	0
S17	S1 OR S2 OR S3 OR S4 OR S5 OR S6 OR S7 OR S8 OR S9 OR S10 OR S11 OR S12 OR S13 OR S14 OR S15 OR S16	Expanders - Apply equivalent subjects<br>Search modes - Boolean/Phrase	Interface - EBSCOhost Research Databases<br>Search Screen - Advanced Search<br>Database - CINAHL Complete	12,737
S18	(MH "Rehabilitation+")	Expanders - Apply equivalent subjects<br>Search modes - Boolean/Phrase	Interface - EBSCOhost Research Databases<br>Search Screen - Advanced Search<br>Database - CINAHL Complete	303,572
S19	(MH "Physical Medicine")	Expanders - Apply equivalent subjects<br>Search modes - Boolean/Phrase	Interface - EBSCOhost Research Databases<br>Search Screen - Advanced Search<br>Database - CINAHL Complete	1,839
S20	TI rehab* OR AB rehab* OR SO rehab*	Expanders - Apply equivalent subjects<br>Search modes - Boolean/Phrase	Interface - EBSCOhost Research Databases<br>Search Screen - Advanced Search<br>Database - CINAHL Complete	164,733
S21	TI telerehab* OR AB telerehab* OR SO telerehab*	Expanders - Apply equivalent subjects<br>Search modes - Boolean/Phrase	Interface - EBSCOhost Research Databases<br>Search Screen - Advanced	511

			Search<br>Database - CINAHL Complete	
S22	TI neurorehab* OR AB neurorehab* OR SO neurorehab*	Expanders - Apply equivalent subjects<br>Search modes - Boolean/Phrase	Interface - EBSCOhost Research Databases<br>Search Screen - Advanced Search<br>Database - CINAHL Complete	5,776
S23	TI ((physiatrist* or physiatry)) OR AB ((physiatrist* or physiatry))	Expanders - Apply equivalent subjects<br>Search modes - Boolean/Phrase	Interface - EBSCOhost Research Databases<br>Search Screen - Advanced Search<br>Database - CINAHL Complete	1,035
S24	(MH "Rehabilitation Centers+")	Expanders - Apply equivalent subjects<br>Search modes - Boolean/Phrase	Interface - EBSCOhost Research Databases<br>Search Screen - Advanced Search<br>Database - CINAHL Complete	8,741
S25	(MH "Occupational Therapy+") or (MH "Occupational Therapists")	Expanders - Apply equivalent subjects<br>Search modes - Boolean/Phrase	Interface - EBSCOhost Research Databases<br>Search Screen - Advanced Search<br>Database - CINAHL Complete	35,565
S26	TI (occupational n1 therap*) OR AB (occupational n1 therap*) OR SO (occupational n1 therap*)	Expanders - Apply equivalent subjects<br>Search modes - Boolean/Phrase	Interface - EBSCOhost Research Databases<br>Search Screen - Advanced Search<br>Database - CINAHL Complete	52,854
S27	(MH "Physical Therapy+") OR (MH "Physical Therapists")	Expanders - Apply equivalent subjects<br>Search modes - Boolean/Phrase	Interface - EBSCOhost Research Databases<br>Search Screen - Advanced Search<br>Database - CINAHL Complete	159,378
S28	TI (physical n1 therap*) OR AB (physical n1 therap*) OR SO (physical n1 therap*)	Expanders - Apply equivalent subjects<br>Search modes - Boolean/Phrase	Interface - EBSCOhost Research Databases<br>Search Screen - Advanced Search<br>Database - CINAHL Complete	48,393
S29	TI physiotherap* OR AB physiotherap* OR SO physiotherap*	Expanders - Apply equivalent subjects<br>Search modes - Boolean/Phrase	Interface - EBSCOhost Research Databases<br>Search Screen - Advanced Search<br>Database - CINAHL Complete	40,087
S30	TI physio-therapist* OR AB physio-therapist* OR SO physio-therapist*	Expanders - Apply equivalent subjects<br>Search modes - Boolean/Phrase	Interface - EBSCOhost Research Databases<br>Search Screen - Advanced Search<br>Database - CINAHL Complete	5

S31	(MH "Speech-Language Pathologists") OR (MH "Speech-Language Pathology Assistants")	Expanders - Apply equivalent subjects<br>Search modes - Boolean/Phrase	Interface - EBSCOhost Research Databases<br>Search Screen - Advanced Search<br>Database - CINAHL Complete	7,836
S32	TI ((speech n2 (therap* or patholog*))) OR AB ((speech n2 (therap* or patholog*))) OR SO ((speech n2 (therap* or patholog*)))	Expanders - Apply equivalent subjects<br>Search modes - Boolean/Phrase	Interface - EBSCOhost Research Databases<br>Search Screen - Advanced Search<br>Database - CINAHL Complete	17,064
S33	(MH "Neuropsychology")	Expanders - Apply equivalent subjects<br>Search modes - Boolean/Phrase	Interface - EBSCOhost Research Databases<br>Search Screen - Advanced Search<br>Database - CINAHL Complete	1,867
S34	TI Neuropsycholog* OR AB Neuropsycholog* OR SO Neuropsycholog*	Expanders - Apply equivalent subjects<br>Search modes - Boolean/Phrase	Interface - EBSCOhost Research Databases<br>Search Screen - Advanced Search<br>Database - CINAHL Complete	17,173
S35	(MH "Dietitians")	Expanders - Apply equivalent subjects<br>Search modes - Boolean/Phrase	Interface - EBSCOhost Research Databases<br>Search Screen - Advanced Search<br>Database - CINAHL Complete	5,366
S36	TI ((Nutritionist* or Dietician*)) OR AB ((Nutritionist* or Dietician*)) OR SO ((Nutritionist* or Dietician*))	Expanders - Apply equivalent subjects<br>Search modes - Boolean/Phrase	Interface - EBSCOhost Research Databases<br>Search Screen - Advanced Search<br>Database - CINAHL Complete	2,594
S37	(MH "Recreational Therapy")	Expanders - Apply equivalent subjects<br>Search modes - Boolean/Phrase	Interface - EBSCOhost Research Databases<br>Search Screen - Advanced Search<br>Database - CINAHL Complete	1,792
S38	(MH "Recreational Therapists")	Expanders - Apply equivalent subjects<br>Search modes - Boolean/Phrase	Interface - EBSCOhost Research Databases<br>Search Screen - Advanced Search<br>Database - CINAHL Complete	180
S39	TI (therap* n1 recreation*) OR AB (therap* n1 recreation*) OR SO (therap* n1 recreation*)	Expanders - Apply equivalent subjects<br>Search modes - Boolean/Phrase	Interface - EBSCOhost Research Databases<br>Search Screen - Advanced Search<br>Database - CINAHL Complete	1,623
S40	TI child life specialist* OR AB child life specialist*	Expanders - Apply equivalent subjects	Interface - EBSCOhost Research Databases	157

	OR SO child life specialist*	Search modes - Boolean/Phrase	Search Screen - Advanced Search<br>Database - CINAHL Complete	
S41	(MH "Play Therapy")	Expanders - Apply equivalent subjects<br>Search modes - Boolean/Phrase	Interface - EBSCOhost Research Databases<br>Search Screen - Advanced Search<br>Database - CINAHL Complete	1,244
S42	TI (play n1 therap*) OR AB (play n1 therap*) OR (play n1 therap*)	Expanders - Apply equivalent subjects<br>Search modes - Boolean/Phrase	Interface - EBSCOhost Research Databases<br>Search Screen - Advanced Search<br>Database - CINAHL Complete	2,257
S43	(MH "Respite Care")	Expanders - Apply equivalent subjects<br>Search modes - Boolean/Phrase	Interface - EBSCOhost Research Databases<br>Search Screen - Advanced Search<br>Database - CINAHL Complete	1,412
S44	TI respite OR AB respite	Expanders - Apply equivalent subjects<br>Search modes - Boolean/Phrase	Interface - EBSCOhost Research Databases<br>Search Screen - Advanced Search<br>Database - CINAHL Complete	1,742
S45	S18 OR S19 OR S20 OR S21 OR S22 OR S23 OR S24 OR S25 OR S26 OR S27 OR S28 OR S29 OR S30 OR S31 OR S32 OR S33 OR S34 OR S35 OR S36 OR S37 OR S38 OR S39 OR S40 OR S41 OR S42 OR S43 OR S44	Expanders - Apply equivalent subjects<br>Search modes - Boolean/Phrase	Interface - EBSCOhost Research Databases<br>Search Screen - Advanced Search<br>Database - CINAHL Complete	520,702
S46	(MH "Brain Injuries+")	Expanders - Apply equivalent subjects<br>Search modes - Boolean/Phrase	Interface - EBSCOhost Research Databases<br>Search Screen - Advanced Search<br>Database - CINAHL Complete	30,448
S47	(MH "Head Injuries")	Expanders - Apply equivalent subjects<br>Search modes - Boolean/Phrase	Interface - EBSCOhost Research Databases<br>Search Screen - Advanced Search<br>Database - CINAHL Complete	7,679
S48	(MH "Brain Concussion+")	Expanders - Apply equivalent subjects<br>Search modes - Boolean/Phrase	Interface - EBSCOhost Research Databases<br>Search Screen - Advanced Search<br>Database - CINAHL Complete	5,571
S49	TI (TBI* OR mTBI*) OR AB (TBI* OR mTBI*)	Expanders - Apply equivalent subjects	Interface - EBSCOhost Research Databases	10,062

		Search modes - Boolean/Phrase	Search Screen - Advanced Search<br>Database - CINAHL Complete	
S50	TI (concuss* or postconcuss*) OR AB (concuss* or postconcuss*)	Expanders - Apply equivalent subjects<br>Search modes - Boolean/Phrase	Interface - EBSCOhost Research Databases<br>Search Screen - Advanced Search<br>Database - CINAHL Complete	5,823
S51	TI (((head* or brain* or cerebr* or crani* or skull* or intracran*) n2 (injur* or trauma* or damag* or wound* or swell* or oedema* or edema* or fracture* or contusion* or pressur*))) OR AB (((head* or brain* or cerebr* or crani* or skull* or intracran*) n2 (injur* or trauma* or damag* or wound* or swell* or oedema* or edema* or fracture* or contusion* or pressur*))) OR SO (((head* or brain* or cerebr* or crani* or skull* or intracran*) n2 (injur* or trauma* or damag* or wound* or swell* or oedema* or edema* or fracture* or contusion* or pressur*)))	Expanders - Apply equivalent subjects<br>Search modes - Boolean/Phrase	Interface - EBSCOhost Research Databases<br>Search Screen - Advanced Search<br>Database - CINAHL Complete	50,589
S52	TI (((brain* or cerebr* or intracerebr* or crani* or intracran* or head* or subdural* or epidural* or extradural*) n1 (haematoma* or hematoma* or hemorrhag* or haemorrhag* or bleed*))) OR AB (((brain* or cerebr* or intracerebr* or crani* or intracran* or head* or subdural* or epidural* or extradural*) n1 (haematoma* or hematoma* or	Expanders - Apply equivalent subjects<br>Search modes - Boolean/Phrase	Interface - EBSCOhost Research Databases<br>Search Screen - Advanced Search<br>Database - CINAHL Complete	12,990

	hemorrhag* or haemorrhag* or bleed*)))			
S53	(MH "Cognition Disorders+")	Expanders - Apply equivalent subjects<br>Search modes - Boolean/Phrase	Interface - EBSCOhost Research Databases<br>Search Screen - Advanced Search<br>Database - CINAHL Complete	33,064
S54	TI (((cogniti* or neurocogniti*) n2 (impair* or dysfunction* or disorder* or declin*))) OR AB (((cogniti* or neurocogniti*) n2 (impair* or dysfunction* or disorder* or declin*)))	Expanders - Apply equivalent subjects<br>Search modes - Boolean/Phrase	Interface - EBSCOhost Research Databases<br>Search Screen - Advanced Search<br>Database - CINAHL Complete	43,126
S55	S46 OR S47 OR S48 OR S49 OR S50 OR S51 OR S52 OR S53 OR S54	Expanders - Apply equivalent subjects<br>Search modes - Boolean/Phrase	Interface - EBSCOhost Research Databases<br>Search Screen - Advanced Search<br>Database - CINAHL Complete	130,656
S56	S17 AND S45	Expanders - Apply equivalent subjects<br>Search modes - Boolean/Phrase	Interface - EBSCOhost Research Databases<br>Search Screen - Advanced Search<br>Database - CINAHL Complete	1,162
S57	S17 AND S55	Expanders - Apply equivalent subjects<br>Search modes - Boolean/Phrase	Interface - EBSCOhost Research Databases<br>Search Screen - Advanced Search<br>Database - CINAHL Complete	179
S58	S56 OR S57	Expanders - Apply equivalent subjects<br>Search modes - Boolean/Phrase	Interface - EBSCOhost Research Databases<br>Search Screen - Advanced Search<br>Database - CINAHL Complete	1,279
S59	S58	Limiters - English Language<br>Expanders - Apply equivalent subjects<br>Search modes - Boolean/Phrase	Interface - EBSCOhost Research Databases<br>Search Screen - Advanced Search<br>Database - CINAHL Complete	1,250
S60	S58 NOT S59	Expanders - Apply equivalent subjects<br>Search modes - Boolean/Phrase	Interface - EBSCOhost Research Databases<br>Search Screen - Advanced Search<br>Database - CINAHL Complete	29

****

Database: Nursing and Allied Health Premium
Platform: Proquest
Last searched: March 24 2022

Search 1:
((NOFT(homeless* OR roofless* OR houseless* OR unsheltered OR ("rough sleepers") OR "rough sleeping") OR NOFT(Marginal* n/3 hous*) OR NOFT(precarious* n/3 hous*) OR NOFT(unstabl* n/3 hous*) OR NOFT(instab* n/3 hous*) OR NOFT(interim* n/3 hous*) OR NOFT(temporary n/3 (liv* or hous*)) OR NOFT((liv* or sleep* or stay or emergenc*) n/3 shelter*) OR NOFT("provisionally accommodat*")) AND (NOFT(rehab* OR telerehab* OR neurorehab* OR physiatrist* OR physiatry OR Neuropsycholog* OR Nutritionist* or Dietician* OR respite) OR PUB(rehab* OR telerehab* OR neurorehab* OR Neuropsycholog* OR Nutritionist* or Dietician* OR Respite) OR NOFT(occupational n/1 therap*) OR PUB(occupational n/1 therap*) OR NOFT(physical n1 therap*) OR PUB(physical n/1 therap*) OR NOFT(physiotherap* or "physio-therap*") OR PUB(physiotherap* or "physio-therap*") OR NOFT(speech n/2 (therap* or patholog*)) OR PUB(speech n/2 (therap* or patholog*)) OR NOFT(therap* n/1 recreation*) OR PUB(therap* n/1 recreation*) OR NOFT("child life specialist*") OR NOFT(play n/1 therap*)))

Search 2:
(NOFT(homeless* OR roofless* OR houseless* OR unsheltered OR "rough sleeper*" OR "rough sleeping") OR NOFT(Marginal* n/3 hous*) OR NOFT(precarious* n/3 hous*) OR NOFT(unstabl* n/3 hous*) OR NOFT(instab* n/3 hous*) OR NOFT(interim* n/3 hous*) OR NOFT(temporary n/3 (liv* or hous*)) OR NOFT((liv* or sleep* or stay or emergenc*) n/3 shelter*) OR NOFT("provisionally accommodat*")) AND (NOFT(TBI* OR mTBI* OR concuss* OR postconcuss*) OR NOFT((head* or brain* or cerebr* or crani* or skull* or intracran*) n/2 (injur* or trauma* or damag* or wound* or swell* or oedema* or edema* or fracture* or contusion* or pressur*)) OR PUB((head* or brain* or cerebr* or crani* or skull* or intracran*) n/2 (injur* or trauma* or damag* or wound* or swell* or oedema* or edema* or fracture* or contusion* or pressur*)) OR NOFT((brain* or cerebr* or intracerebr* or crani* or intracran* or head* or subdural* or epidural* or extradural*) n/1 (haematoma* or hematoma* or hemorrhag* or haemorrhag* or bleed*)) OR NOFT((cogniti* or neurocogniti*) n/2 (impair* or dysfunction* or disorder* or declin*)))

Total: 326 citations

****

Database: Applied Social Sciences Index & Abstracts (ASSIA)
Platform: Proquest
Last searched: March 24 2022

Search 1:
((NOFT(homeless* OR roofless* OR houseless* OR unsheltered OR ("rough sleepers") OR "rough sleeping") OR NOFT(Marginal* n/3 hous*) OR NOFT(precarious* n/3 hous*) OR NOFT(unstabl* n/3 hous*) OR NOFT(instab* n/3 hous*) OR NOFT(interim* n/3 hous*) OR NOFT(temporary n/3 (liv* or hous*)) OR NOFT((liv* or sleep* or stay or emergenc*) n/3 shelter*) OR NOFT("provisionally accommodat*")) AND (NOFT(rehab* OR telerehab* OR neurorehab* OR physiatrist* OR physiatry OR Neuropsycholog* OR Nutritionist* or Dietician* OR respite) OR PUB(rehab* OR telerehab* OR neurorehab* OR Neuropsycholog* OR Nutritionist* or Dietician* OR Respite) OR NOFT(occupational n/1 therap*) OR PUB(occupational n/1 therap*) OR NOFT(physical n1 therap*) OR PUB(physical n/1 therap*) OR NOFT(physiotherap* or "physio-therap*") OR PUB(physiotherap* or "physio-therap*") OR NOFT(speech n/2 (therap* or patholog*)) OR PUB(speech n/2 (therap* or patholog*)) OR

NOFT(therap* n/1 recreation*) OR PUB(therap* n/1 recreation*) OR NOFT("child life specialist*") OR NOFT(play n/1 therap*)))

Search 2:
(NOFT(homeless* OR roofless* OR houseless* OR unsheltered OR "rough sleeper*" OR "rough sleeping") OR NOFT(Marginal* n/3 hous*) OR NOFT(precarious* n/3 hous*) OR NOFT(unstabl* n/3 hous*) OR NOFT(instab* n/3 hous*) OR NOFT(interim* n/3 hous*) OR NOFT(temporary n/3 (liv* or hous*)) OR NOFT((liv* or sleep* or stay or emergenc*) n/3 shelter*) OR NOFT("provisionally accommodat*")) AND (NOFT(TBI* OR mTBI* OR concuss* OR postconcuss*) OR NOFT((head* or brain* or cerebr* or crani* or skull* or intracran*) n/2 (injur* or trauma* or damag* or wound* or swell* or oedema* or edema* or fracture* or contusion* or pressur*)) OR PUB((head* or brain* or cerebr* or crani* or skull* or intracran*) n/2 (injur* or trauma* or damag* or wound* or swell* or oedema* or edema* or fracture* or contusion* or pressur*)) OR NOFT((brain* or cerebr* or intracerebr* or crani* or intracran* or head* or subdural* or epidural* or extradural*) n/1 (haematoma* or hematoma* or hemorrhag* or haemorrhag* or bleed*)) OR NOFT((cogniti* or neurocogniti*) n/2 (impair* or dysfunction* or disorder* or declin*)))

Total: 184 citations

Grey literature search

Websites of Brain Injury, Housing, and Rehabilitation Organizations that were searched, and the date it was searched:

1. American Academy of Physical Medicine and Rehabilitation (May 6, 2021; May 17, 2021)
2. Brain injury Canada (May 6, 2021; May 20, 2021)
3. Calgary Homeless Foundation (May 6, 2021)
4. Canadian Alliance to End Homelessness (May 6, 2021; May 25, 2021)
5. Canadian Housing First Toolkit (May 6, 2021; June 1, 2021)
6. Canadian Institute for Military and Veteran Health Research (June 10, 2021; July 21, 2021)
7. Centre for Urban Health Solutions (May 21, 2021; June 2, 2021)
8. Cochrane Methods Equity Homeless Health Guidelines (May 20, 2021; June 3, 2021)
9. Evidence Exchange Network for Mental Health Addictions (June 1, 2021)
10. Mental Health Commission of Canada (May 6, 2021; June 1, 2021)
11. Model Systems Knowledge Translation Center (May 14, 2021)
12. National Association of State Head Injury (May 14, 2021)
13. National Association of Veterans' Research and Education Foundations (June 10, 2021; July 21, 2021)
14. National Health Care for the Homeless Council (May 14, 2021; June 1, 2021)
15. National Institute on Disability, Independent Living and Rehabilitation Research (NIDILRR) (September 25, 2021)
16. Ruff Institute of Global Homelessness (May 12, 2021; May 14, 2021; June 1, 2021)
17. The Center for Brain Injury Research and Training (May 14, 2021; June 1, 2021)
18. The Homeless Hub (June 1, 2021)
19. The National Rehabilitation Information Center (September 25, 2021)
20. Toronto Alliance to End Homelessness (June 21, 2021)
21. Toronto Mental Health and Addictions Supportive Housing Network (May 21, 2021; June 21, 2021)
22. US department of Veterans' affairs (July 5, 2021; July 21, 2021)
23. Veterans & Families Research Hub (June 11, 2021; July 21, 2021)
24. Veterans Affairs Canada (June 10, 2021; July 1, 2021)
25. Wellesley Institute (May 21, 2021; June 21, 2021)
26. Women's National Housing and Homelessness Network (May 6, 2021; June 1, 2021)

Keywords:

The following keywords were used to search for relevant grey literature reports:

- Concept A: homeless, housing, unsheltered, unhoused
- Concept B: rehabilitation, neuropsychologists, nurse, nutritionist, occupational therapist, physiatrist, physician, physiotherapist, psychologist, psychometrist, social worker, speech language pathologist, therapeutic recreationist
- Concept C: traumatic brain injury, brain injury; cognitive impairment, disability, trauma
