# Supplementary material for: Rehabilitation among individuals experiencing homelessness and traumatic brain injury: A scoping review
Source: Front Med (Lausanne). 2022 Nov 11;9:916602. doi: 10.3389/fmed.2022.916602 (PMC9692012; doi:10.3389/fmed.2022.916602)
Supplement: Supplementary File 2 — Charting table. [file Data_Sheet_2.pdf]

Study (Author, Year, Country)	Study Design & Objective	Study Sample[a]	TBI Status	Homelessness Status	Rehabilitation Intervention, Team, Outcome	TBI-Specific Facilitators, Barriers, Gaps
Adair et al., 2017<br><br>Canada	RCT<br><br>To use generalized growth mixture modeling to examine outcome trajectories of housing stability among individuals who are homeless	N=2,140<br>- Age [Years]: 35-54 (57.0%)<br>- Gender [Males]: 67.0%<br>- Ethnocultural status: Aboriginal (22.0%)<br>- Country of birth: Canada (81.0%)<br>- Education: Not completed high school (55.0%)<br>- Employment: Unemployed (93.0%), <$300 CAD in the prior mos (24.0%)	- Hx of TBI (66.0%)	- Individuals experiencing unstable housing, including living on the street or temporary residencies, shelters, crisis units, or institutions<br>- Absolutely homeless (82.0%)	Intervention:<br>- At Home/Chez Soi trial: Immediate receipt of housing support and resources without requisites of sobriety<br>-Randomly assigned to HF or TAU; those w/high needs received ACT and those w/ moderate needs received ICM<br><br>Rehabilitation Team:<br>- Case managers, psychiatrists, nurses, and peer support workers<br><br>Outcome:<br>- Housing stability (days in stable housing)<br>- 74% of HF and 43% of TAU were in table housing after 24 mos of follow-up<br>- Gender, age, prior month income, Aboriginal status, total time homeless, previous hospitalizations, overall health, psychiatric sxs, and comorbidity distinguished different trajectories	NR
Bean et al., 2013<br><br>United States	Before-After (Pre-Post) Studies w/No Control Group<br><br>To assess the impact of Project H3, an HF, harm-reduction and peer-support intervention for individuals who are homeless and medically vulnerable	Move-in and 6-mos sample N=20:<br>- Age [Mean ± SD, Years]: 53.85 ± 7.03<br>- Gender [Males]: 90.0%<br>- Race: White (60.0%), Black (20.0%), Native American (15.0%), Latino (5.0%)<br>- Ever been to jail: 80.0%<br>- Ever been to prison: 40.0%<br><br>6-month and 12-mos sample N=180:<br>- Age [Mean ± SD, Years]: 56.06 ± 6.04<br>- Gender [Males]: 72.2%<br>- Race: White (55.6%), Black (22.2%), Native American (16.7%), Latino (5.6%)	- Move-in and 6-mos (36.8%)<br>- 6-mos and 12-mos (22.2%)	- Individuals who were experiencing homelessness and who were medically vulnerable<br>- Years homeless [Mean ± SD]: 9.18 ± 7.26 (move-in and 6 mos); 11.91 ± 9.89 (6-mos and 12-mos)	Intervention:<br>- Incorporates HF, harm-reduction, and peer support approaches to provide stable housing individuals who were medically vulnerable and homeless<br><br>Outcome:<br>- 98% remained in stable housing after 12 mos<br>- Significant increase in access to and use of planned healthcare services and QoL, involvement with criminal justice system	NR

Study (Author, Year, Country)	Study Design & Objective	Study Sample[a]	TBI Status	Homelessness Status	Rehabilitation Intervention, Team, Outcome	TBI-Specific Facilitators, Barriers, Gaps
		- Ever been to jail: 77.8%<br>- Ever been to prison: 17.6%				
Brocht et al., 2020<br><br>United States	Case Study<br><br>To describe the experiences of a medical respite program for individuals experiencing TBI and homelessness and the importance of screening, staff education and access to on-going services	- Male/Man (Case study referred to this individual as "he/his")<br>- Employed as engineering assistant for several years in his 20s and 30s<br>- Divorced	- Sustained TBI during car accident in his 20s	- Living in tent encampment for 20 yrs; never stayed in shelter before	Intervention:<br>- The Baltimore Health Care for the Homeless Medical Respite provides acute and post-acute medical care for individuals who are homeless and recovering from a physical illness/injury<br>-Services included nursing, care coordination, education, medication reconciliation; community health worker services (linkage to care and transportation); social work services (pyschosocial evaluation, counseling, referral and case management); occupational therapy (functional skill assessment and development)<br><br>Rehabilitation Team:<br>- OTs, SWs, registered nurse, and community health worker<br><br>Outcome:<br>- Remains engaged in interdisciplinary services at the outpatient clinic at Baltimore Health Care for the Homeless<br>- Continues to seek employment<br>- Is an effective advocate for people experiencing homelessness and is involved in many community activities	Facilitators:<br>- TBI screening to improve tx of cognitive and functional impairments<br>- Educating respite staff on working w/clients w/TBI to inform them of limitations and possible modifications<br>- Modification of the physical environment<br>- Linkages to ongoing rehabilitation services<br><br>Gaps:<br>- Care differences (i.e., type and quality) following a TBI b/t homeless and non-homeless patients<br>- Factors that influence treatment-seeking for those experiencing homelessness and TBI<br>- Effective strategies and interventions for engaging individuals experiencing homelessness and TBI w/in healthcare and housing systems<br>- Educational resources for screening and treating patients w/TBI in homeless service settings
Chung et al., 2018<br><br>Canada	RCT<br><br>To compare the effect of HF between older (> 50 years old) and younger	≥50 yrs old (N = 470)<br>- Age [Mean ± SD, Years]: 55.8 ± 4.9<br>- Gender [Male]: 70.6%<br>- Racial, ethnic, or cultural identity: Aboriginal (11.9%),	- >50 years old (62.1%)<br>- 18-49 year old (67.4%)	Housing Status:<br>- Absolutely homeless: ≥50 yrs old (82.1%), 18-49 yrs old (81.4%)	Intervention:<br>- At Home/Chez Soi: Immediate receipt of housing support and additional services and resources without requisites of sobriety or tx adherence	NR

Study (Author, Year, Country)	Study Design & Objective	Study Sample[a]	TBI Status	Homelessness Status	Rehabilitation Intervention, Team, Outcome	TBI-Specific Facilitators, Barriers, Gaps
	(18-49 years old) homeless adults w/ mental illness	Ethno-racial (19.8%), White (68.3%)<br>- Education: <high school (46.0%), completed high school only (21.0%), some post-secondary school (33.0%)<br>- Income ($CAD Mean ± SD): 740.1 ± 582.9<br>- Arrests in past 6 mos: 16.1%<br><br><u>18-49 yrs old (N=1678)</u><br>- Age [Mean ± SD, Years]: 36.8 ± 8.7<br>- Gender [Male]: 66.3%<br>- Racial, ethnic, or cultural identity: Aboriginal (24.4%), Ethno-racial (26.2%), White (49.5%)<br>- Education: < high school (58.1%), completed high school only (18.2%), some post-secondary school (23.6%)<br>- Income ($CAD Mean ± SD): 663.7 ± 707.7<br>- Arrests in the past 6 mos: 29.9%		- Precariously housed: ≥50 yrs old (17.9%), 18-49 yrs old (18.6%)<br>-Age at first homelessness [Mean ± SD, Years]: ≥50 yrs old (44.4 ± 13.8); 18-49 yrs old (27.6 ± 10.4)<br><br>Lifetime duration of homelessness [Mean ± SD, Years]: ≥50 yrs old (5.5 ± 7.0); 18-49 yrs old (4.6 ± 5.3)	- Randomly assigned to HF or TAU; those w/high needs received ACT and those w/moderate needs received ICM<br><br><u>Rehabilitation Team:</u><br>- Case managers, psychiatrists, nurses, and peer support workers<br><br><u>Outcome:</u><br>- HF significantly improved housing stability among older and youth homeless adults w/mental illness<br>- super mental health and QoL outcome in older vs. younger homeless adults at 24 mos	
Fine et al., 2021<br><br>United States	Observational<br><br>To assess patient-reported experiences with a mobile health program	<u>N=91</u><br>- Age [Mean ± SD, Years]: 39.5 ± 12.3<br>- Sex: Males (70.3%), females (26.4%), other (3.30%)<br>- Race: Non-Hispanic white (58.2%), non-Hispanic black (19.8%), Hispanic/Latinx (16.5%), other (5.5%)	NR	Housing status:<br>- Housed (19.8%), shelter (22.0%), unsheltered (26.4%), doubled-up (24.2%), other (7.7%)	<u>Intervention:</u><br>- Mobile health outreach program: harm reduction services, addiction tx, and primary care provide in a mobile unit to four opioid overdose hotspots in Boston, Massachusetts consistently on the same day at the same time each week<br><br><u>Rehabilitation team:</u><br>- Addiction medicine clinicians, primary care clinicians, public health advocates, harm reduction specialists, medical case manager<br><br><u>Outcome:</u>	NR

Study (Author, Year, Country)	Study Design & Objective	Study Sample[a]	TBI Status	Homelessness Status	Rehabilitation Intervention, Team, Outcome	TBI-Specific Facilitators, Barriers, Gaps
					- Top 3 cited service that attracted individuals to the program were provision of new needles (33.0%), food/drink (27.5%), and buprenorphine prescriptions (25.3%)<br>- 98.9% felt trusted and respected by program staff, 97.8% reported program fit their healthcare needs<br>- Most frequently cited recommendation was for adding behavioural health services, hepatitis C tx, and more information about hours and locations of program<br>- Majority reported mobile program was better than traditional office-based care	
Gargaro et., 2016<br><br>Canada	Observational<br><br>To determine whether users of an ACT Team for homeless individuals w/ serious MHSU problems had a hx of TBI	N= 48<br>- Age [Mean ± SD, Years]: 43.40 ± 13.03<br>- Sex [Male]: 69.0%<br>- Education: Not completed high school (33.0%)	- 56.0% screened positive for lifetime hx of TBI (OSU-TBI-ID)	- Individuals who were homeless	Intervention:<br>- ACT Team<br><br>Rehabilitation Team:<br>- Nurse case managers, vocational case manager, addictions case manager, mental health case managers, peer support specialists, and psychiatrists<br><br>Outcome:<br>- 56.0% of participants screened positive for lifetime reported TBI<br>- Clients w/hx of TBI were more likely to be using multiple substances and were more likely to have and be bothered by family and emotional problems than clients without TBI	Barriers:<br>- Lack of understanding from ACT Team clinicians on importance of TBI hx on psychiatric care<br>- Reliability of self-report OSU TBI-ID screener<br>- Participants w/TBI hx more likely to use substances, which could exacerbate functional limitations<br>- Participants w/TBI hx were more likely to report cognitive impairments which may impact their ability to engage in rehabilitation<br>- Standard mental health practices may be a barrier to service engagement for TBI patients and mental health providers may not be aware of patients' TBI hx and may not modify strategies to support cognitive deficits<br><br>Gaps:<br>- Evaluate which strategies and accommodations will

Study (Author, Year, Country)	Study Design & Objective	Study Sample[a]	TBI Status	Homelessness Status	Rehabilitation Intervention, Team, Outcome	TBI-Specific Facilitators, Barriers, Gaps
						maximize tx engagement and positive outcomes for TBI patients who are homeless
Goering et al., 2014<br><br>Canada	RCT<br><br>To systematically compare the HF intervention to existing approaches (i.e., TAU) in five Canadian cities (Vancouver, Winnipeg, Toronto, Montreal and Moncton)	N= 1158<br>- Age [Years]: <34 (33.0%), 35-54 (57.0%), ≥55 (10.0%)<br>- Gender: Males (67.0%), "Other" (1.0%)<br>- Ethnicity: Aboriginal (22.0%), belonged to other ethno-cultural groups (25.0%)<br>- Relationship status: Single, separated, divorced, or widowed (94%)<br>- Education Level: Did not complete high school (55.0%)<br>- Employment Status: Unemployed at study entry (93.0%), worked steadily in the past (66.0%), veteran (4.0%)<br>- Prior justice system involvement: 36.0%	- Hx of ≥1 TBI (66.0%)	- Absolutely homeless (82.0%)<br>- Precarious living situations (18.0%)<br><br>- Average total time homeless: <5 yrs<br>- Homelessness before age 25 yrs: >40%	Intervention:<br>- At Home/Chez Soi trial: Immediately receiving housing support and additional services and resources without requisites of sobriety or tx adherence<br>- Participants were randomly assigned to HF or TAU<br>- Participants w/high needs received ACT and those w/moderate needs received ICM<br><br>Rehabilitation Team:<br>- Case managers, psychiatrists, nurses, and peer specialist<br><br>Outcome:<br>- Housing stability outcomes: 62.0% of HF participants were housed for all of the last 6 mos of the study, 22.0% some of the time, and 16.0% none of the time	NR
Gutman et al., 2004<br><br>United States	Before-After (Pre-Post) Studies with no Control Group<br><br>To evaluate the effectiveness and user acceptability of an intervention for women w/ disabilities who are experiencing (a) domestic violence, (b)	N= 26<br>- Age [Mean, Years]: 44<br>- Gender [Women]: 100.0%<br>- Race/Ethnicity: African-American (42.0%), Caucasian (35.0%), Latino (15.0%), Indian (8.0%)<br>- Marital status: Single (46.0%), married (19.0%), divorced (15.0%), separated (12.0%), lived w/partner (8.0%)<br>- Education: Completed high school (38.0%), completed some college courses but had not earned a degree (27.0%), started but did not complete high school (15.0%), earned	- 19.0% of women who reported domestic abuse had a TBI dx	- 38.0% of participants were homeless<br>- 12% reported becoming homeless after experiencing domestic abuse	Intervention:<br>- Addressed the following participant identified needs: (a) safety planning; (b) drug and alcohol awareness; (c) safe sex education; (d) assertiveness and advocacy training; (e) anger management; (f) stress management; (g) boundary establishment; (h) vocational and educational training; (i) money management; (j) housing support; (k) leisure exploration; (l) hygiene, medication, and nutrition<br><br>Rehabilitation Team:<br>- Delivered by 4 graduate (professional level) OT students and supervised by 2 OTs<br><br>Outcome:	Facilitators:<br>- Intervention tailored to address participants' cognitive deficits; breaking down goals into several smaller tasks matched to functional level; compensatory strategies, repeated practice, and adaptive devices supported participants in completing activities<br>- Intervention design acknowledged that women experiencing domestic abuse and who may have TBI resulting from abuse, require

Study (Author, Year, Country)	Study Design & Objective	Study Sample[a]	TBI Status	Homelessness Status	Rehabilitation Intervention, Team, Outcome	TBI-Specific Facilitators, Barriers, Gaps
	homelessness or (c) both domestic violence and homelessness	college degrees (12.0%), completed elementary special education programs but did not receive any further education (8.0%)<br>- Previously experienced/ presently experiencing domestic violence: 62.0%			- GAS scores were developed for each participant based on ability to achieve desired goals<br>- 81.0% of participants earned GAS scores above 50, demonstrating that they achieved their desired goals<br>- 99.0% of participants reported a high degree of satisfaction with the intervention	time to make small changes and change is not linear<br><br><u>Gaps:</u><br>- Prevalence of TBI resulting from domestic abuse<br>- Develop and evaluate tx tailored for women with cognitive impairment
Kozloff et al., 2016<br><br>Canada	RCT<br><br>To examine and compare demographic, clinical and service use characteristics of homeless youth with mental illness participating in the HF trial	<u>≤24 yrs (N=164)</u><br>- Age [Mean ± SD, Years]: 21.6 ± 1.5<br>- Gender [Non-male gender]: 39.0%<br>- Racial, ethnic, or cultural identity: Aboriginal (27.0%), Ethno-racial minority (34.0%), White (39.0%)<br>- Monthly income ($CAD, Mean ± SD): 563.33 ± 937.8<br>- Education: Did not complete high school (76.0%), completed high school only (15.0%), some postsecondary school (9.0%)<br>- Employment: Currently employed (4.0%)<br>- Arrested in past 6 months: 37.0%<br><br><u>> 24 yrs (N=2091)</u><br>- Age [Mean ± SD, Years]: 42.4 ± 10.2<br>- Gender [Non-male gender]: 32.0%<br>- Racial, ethnic, or cultural identity: Aboriginal (21.0%), Ethno-racial minority (24.0%), White (55.0%)<br>- ($CAD, Mean ± SD): 698.73 ± 657.9	Lifetime TBI:<br>- ≤24 yrs (61.0%)<br>- >24 yrs (66.0%)	Current housing status:<br>- Absolutely homelessness: 87.0% (≤24 yrs); 81.0% (>24 yrs)<br>- Precariously housed: 13% (≤24 yrs); 19% (>24 yrs)<br><br>Lifetime duration of homelessness [Mean ± SD, Years]: 26.1 ± 26.3 (≤24 yrs); 60.7 ± 71.5 (>24 yrs)<br><br>Age at first homelessness [Mean ± SD, Years]: 18.1 ± 3.2 (≤24 yrs); 32.2 ± 13.1 (>24 yrs)	<u>Intervention:</u><br>- At Home/Chez Soi trial: Immediate housing support and additional services and resources without requisites of sobriety or tx adherence<br>- Participants were randomly assigned to HF or TAU<br>- Participants with high needs received ACT and those with moderate needs received ICM<br><br><u>Rehabilitation Team:</u><br>- Case managers, psychiatrists, nurses, and peer support workers<br><br><u>Outcome:</u><br>- Youth's trajectories to homelessness and service needs are distinct from adults	<u>Gap</u>: Service provider training and collaboration w/specialized services on TBI, given the cognitive, behavioural, and emotional sequalae associated w/TBI

Study (Author, Year, Country)	Study Design & Objective	Study Sample[a]	TBI Status	Homelessness Status	Rehabilitation Intervention, Team, Outcome	TBI-Specific Facilitators, Barriers, Gaps
		- Education: Did not complete high school (54.0%), completed high school only (19.0%), some postsecondary school (27.0%)<br>- Employment: Currently employed (3.0%)<br>- Arrested in past 6 months: 27.0%				
Langi et al., 2017<br><br>United States	Observational<br><br>To examine the association b/t residential arrangement and VR outcomes among adults w/ disabilities participating in a state-run VR program	Homeless/shelter (N=567):<br>- Age [Years]: 18-30 (21.5%), 31-40 (22.2%), 41-50 (33.7%), 51-65 (22.6%)<br>- Gender [Female]: 40.0%<br>- Ethnicity: Black (39.3%), Hispanic (5.1%)<br>- Education: ≤Secondary or special education (26.3%), high school graduate/ equivalent (42.5%), >high school (31.2%)	- 1.1% of the sample had a TBI	- Residential arrangement at program intake: homeless/ shelter	Intervention:<br>- The VR program provides individualized and supportive services to persons with disabilities to assist with attainment of employment and other rehabilitation goals<br><br>Outcome:<br>- Compared to individuals living in private residence, those living in homeless/shelter were significantly less likely to be rehabilitated	NR
McHugo et al., 2021<br><br>United States	Observational<br><br>To determine whether integrated, algorithm-driven SUD tx can reduce substance use and improve community function in individuals w/ severe, chronic impairments	N= 305<br>- Age [Mean ± SD, Years]: 44.99 ± 9.31<br>- Sex [Females]: 60.7%<br>- Race: African American (94.4%)<br>- Number of lifetime times in jail/prison [Mean ± SD]: 5.72 ± 13.89<br>- Number of lifetime arrests, non-violent crimes [Mean ± SD]: 4.62 ± 10.96<br>- Number of lifetime arrests, violent crimes [Mean ± SD]: 1.24 ± 3.07	- 79.9% (OSU-TBI-ID)	- Number of lifetime months homelessness [Mean ± SD]: 39.51 ± 67.03	Intervention:<br>- Dual Diagnosis Case Management: Interested participants were eligible for an evidence-based group intervention to reduce alcohol or drug use, contingency management to reduce drug use, or naltrexone to reduce alcohol use<br>- Participants who developed abstinence received relapse prevention of their choice (supported employment, Dual Diagnosis Case Management, Alcoholics or Narcotics Anonymous)<br>- Participants not interested in SUD interventions continued to receive Dual Diagnosis Case Management<br><br>Rehabilitation Team:<br>- Clinicians (case managers)	NR

Study (Author, Year, Country)	Study Design & Objective	Study Sample[a]	TBI Status	Homelessness Status	Rehabilitation Intervention, Team, Outcome	TBI-Specific Facilitators, Barriers, Gaps
					Outcome:<br>- 2/3 of participants remained in tx for 2 yrs, ¼ of completers achieved ≥3 mos of abstinence	
Mejia-Lancheros et al., 2020<br><br>Canada	RCT<br><br>To assess the effectiveness of a HF intervention on incidence of violence-related TBI in adults experiencing homelessness and mental illness	N= 381<br>- Age [Mean ± SD, Years]: 40.6 ± 11.7<br>- Gender [Men]: 68.0%<br>- Ethno-racial group: Non-white (57.2%)<br>- Education level: Completed up to high school (64.9%)	- 100% (Self-reported TBI determined through face-to-face interviews using a validated survey instrument)	- Absolutely homeless or precariously housed<br>- Lifetime Homeless: <3 yrs (44.7%); ≥3 yrs (55.3%)	Intervention:<br>- At Home/Chez Soi trial: Immediately receiving housing support and additional services and resources without requisites of sobriety or tx adherence<br>- Participants were randomly assigned to HF or TAU; participants w/high needs received ACT and those w/moderate needs received ICM<br><br>Rehabilitation Team:<br>- Case managers, psychiatrists, nurses, and peer support workers<br><br>Outcome:<br>- HF participants, compared to TAU, had significantly lower # of physical violence-related TBI events	Barriers:<br>- Reducing the risk of violence-related TBI is difficult due to intersecting factors such as substance use, poverty and exclusion<br><br>Gaps:<br>- Integrate community-based, mental health, and harm reduction support services such as HF to reduce exposure to TBI and improve housing stability<br>- Studies that use clinical and administrative data to identify TBI and inform the development of interventions to reduce TBI sequelae among individuals experiencing homelessness<br>- Examining the impact of HF in preventing TBI to prevent and reduce poor health outcomes
Merryma-n & Synovec, 2020<br><br>United States	Qualitative study<br><br>To understand the perceptions of providers on the utility of a new occupational therapy intervention for homeless	NR	- 51.0% (head trauma)	- Study setting: FQHC providing comprehensive primary and integrated health care to individuals who are low income and/or experiencing homelessness	Intervention:<br>- Occupational therapy service at FQHC: Completion of cognitive and functional evaluations, documented results and provided recommendations<br><br>Rehabilitation Team:<br>- Multidisciplinary team including physicians, nurses, SWs, case managers and registered OTs<br><br>Outcome:	Facilitators:<br>- Alternative view of function through functional and informal assessments, specifically for complex clients such as TBI that informed tx and engagement w/clients

Study (Author, Year, Country)	Study Design & Objective	Study Sample[a]	TBI Status	Homelessness Status	Rehabilitation Intervention, Team, Outcome	TBI-Specific Facilitators, Barriers, Gaps
	adults at a FQHC				- OTs provided important view of functional capacity and understanding of complex impairments<br>- Integrating OT increased access to immediate consultation<br>- OT enriched client services by supporting subsequent tx decisions<br>- Providers valued written and verbal recommendation	
Schiff J. 2014<br><br>Canada	Observational/ Qualitative<br><br>To examine if four HF programs established independently of a research project, and operating under different principles of service delivery, would serve the same types of clients as those under a focused HF study such as the At Home/Chez Soi study conducted by the Mental Health Commission of Canada	Homebase Program N=50<br>- Age [Years]: ≤34 (24.0%), 35-45 (39.0%), ≥55 (37.0%)<br>- Gender [Males]: 78.0%<br>- Country of Birth: Canada (100%)<br>- Ethnic Status: Aboriginal (30.0%), other ethno-cultural (11.0%)<br>- Education: <high school (76.0%), high school (12.0%), any post-secondary (12.0%)<br>- Marital status: Single, never married (96.0%), married or common-law (4.0%)<br><br>Houselink Program N=74<br>- Age [Years]: ≤34 (6.0%), 35-45 (56.0%), ≥55 (38.0%)<br>- Gender [Male]: 53.0%<br>- Ethnic Status: Aboriginal (5.0%), Other (35.0%)<br>- Education: <high school (47.0%), high school (1.0%), any post-secondary (52.0%)<br>- Marital status: Single, never married (80.0%), other (20.0%)<br><br>Pathways Calgary N=75<br>- Age [Years]: ≤34 (23.0%), 35-45 (61.0%), ≥55 (16.0%)<br>- Gender [Male]: 77.0%	- Homebase (60.0%); Houselink (36.0%); Pathways Calgary program (37.0%); Pathways Edmonton program (45.0%)	Homebase Program:<br>- Living arrangement prior to program entry: Shelter (41.0%), Institution (26.0%), Doubling up – friends and family (19.0%), transitional housing & unspecified (14.0%)<br>- Longest period of homelessness [mos]: 34<br><br>Houselink Program: NR<br><br>Pathways Calgary:<br>- Living arrangement prior to program entry: Shelter (45.0%), Institution (36.0%),	Intervention:<br>- Homebase Program:<br>- ICM model to provide stable housing<br>- About 13- 17 clients are assigned to each case manager, who determines the level of need and linkage to support services for clients<br>- Case managers meet with clients twice a week, in their housing apartments<br><br>- Houselink Program:<br>- Housed in agency owned units to help address challenges of daily living<br>- Gears all of its activities to assuring that members are securely and safely housed and are then provided with social and recreational activities, personal development, and vocational, educational and employment opportunities<br><br>- Pathways Calgary:<br>- Includes a full medical clinic, counseling and activity rooms and space for vocationally oriented activities.<br>- The ACT approach offers 24/7 supports services<br><br>- Pathways Edmonton:<br>-Activities consist primarily of individual support sessions, most frequently as outreach calls or home visits to clients	NR

Study (Author, Year, Country)	Study Design & Objective	Study Sample[a]	TBI Status	Homelessness Status	Rehabilitation Intervention, Team, Outcome	TBI-Specific Facilitators, Barriers, Gaps
		- Ethnic Status: Aboriginal (13.0%), Other (16.0%)<br>- Education: <high school (67.0%), high school (19.0%), any post-secondary (14.0%)<br>- Marital status: Single, never married (68.0%), other (32.0%)<br><br>Pathways Edmonton N=75<br>- Age [Years]: ≤34 (21.0%), 35-45 (66.0%), ≥55 (13.0%)<br>- Gender [Male]: 52.0%<br>- Ethnic Status: Aboriginal (35.0%)<br>- Education: <high school (26.0%), high school (48.0%), any post-secondary (26.0%)<br>- Marital status: Single, never married (62.0%), married or common-law (4.0%, other (34.0%)		Doubling up-friends and family (4.0%), transitional housing & unspecified (15.0%)<br>- Chronically homeless at entry: 99.0%<br>- Spent ≥1 night in shelter in last 6 mos: 89.0%<br><br>Pathways Edmonton:<br>- Living arrangement prior to program entry: Shelter (59.0%), institutions (24.0%), transitional housing & unspecified (23.0%)<br>- Stayed in a shelter ≥1 night prior to entry: 59.0%<br>- Chronically homeless at entry: 70.0%<br>- Average length of homelessness [mos]: 66	- Clients are seen at the office for medication and psychiatric consultation appointments<br><br>Rehabilitation Team:<br>- Homebase Program: case managers<br>- Houselink Program: SWs, recreation therapy and rehabilitation workers<br>- Pathways Calgary: physicians, psychiatrists, nurse practitioner, registered nurses, SWs, mental health case managers, recreation therapist, and nutritionist<br>- Pathways Edmonton: part-time psychiatrist, medical doctor, mental health and substance abuse outreach workers, OT	
Stergiopoulos et al., 2014	RCT<br><br>To systematically	N=575<br>- Age [Years]: ≤34 (36.0%), 35-54 years (54.0%), ≥55 (10.0%)	- 54.0%	- Absolute homelessness (93%) or	Intervention:<br>- At Home/Chez Soi trial: Immediately receiving housing support and additional	NR

Study (Author, Year, Country)	Study Design & Objective	Study Sample[a]	TBI Status	Homelessness Status	Rehabilitation Intervention, Team, Outcome	TBI-Specific Facilitators, Barriers, Gaps
Canada	compare HF intervention to existing approaches (i.e., TAU) at the Toronto study site	- Gender: Male (68.0%), Other (2.0%)<br>- Ethnic Status: Aboriginal (5.0%), other ethno-cultural (59.0%)<br>- Country of birth: Canada (54.0%), other (46.0%)<br>- Marital status: Single, never married (70.0%), married or common-law (4.0%), other (26.0%)<br>- Education: <high school (49.0%), high school (19.0%), any post-secondary (12.0%)<br>- Prior monthly income <$300: 28%<br>- Currently unemployed: 95%<br>- Justice system involvement (arrested >once, incarcerated or served probation in prior 6 mos): 38.0%<br>- Justice system involvement type detained by police (25.0%), held in police cell 24 hours or less (19.0%), arrested (29.0%), court appearance (34.0%), attended a justice service program (15.0%)		precariously housed (7%)	services and resources without requisites of sobriety or tx adherence<br>- Participants were randomly assigned to HF or TAU; those w/high needs received ACT and those w/moderate needs received ICM<br><br>Rehabilitation Team:<br>- Case managers, psychiatrists, nurses, and peer support workers<br><br>Outcome:<br>- Housing stability outcomes: 72.0% of HF participants were housed for all of the last six months of the study, 16.0% some of the time, and 12.0% none of the time	
Synovec & Berry, 2019<br><br>United States	Observational<br><br>Pilot training for mental health providers to address the issues of brain injury	NR	- OSU TBI-ID (84.0%)	- The study setting was a FQHC, which provides comprehensive primary and integrated health care to individuals who are low income and/or experiencing homelessness	Intervention:<br>- The study included (1) implementation of a TBI screening tool (OSU TBI-ID) during OT evaluation for all eligible participants; (2) 5-month provider training program focused on instrument use, SMART goal writing and strategies to address cognitive deficits associated with TBI<br><br>Rehabilitation Team:<br>- Multidisciplinary team including physicians, nurses, SWs, case managers, and registered OTs<br><br>Outcome:	Facilitators:<br>- Providers learning specific tools to identify a hx of TBI is beneficial in determining functional implications for planning and goal setting<br>- Structured training sessions on specific strategies to address common cognitive needs of those with TBI are also beneficial in increasing the efficacy of interventions.<br><br>Gaps

Study (Author, Year, Country)	Study Design & Objective	Study Sample[a]	TBI Status	Homelessness Status	Rehabilitation Intervention, Team, Outcome	TBI-Specific Facilitators, Barriers, Gaps
					- Providers reported an increased in self-efficacy in using the OSU TBI-ID to screen for TBI and the WHO-DAS tool to assess goal areas for tx<br>- Providers demonstrated an increase in knowledge and use of SMART goals<br>- 50.0% reported that SMART goals were useful in addressing needs of TBI patients<br>- Providers reported an increase in knowledge and use of strategies that were effective for TBI patients	-Need for screening and training programs to support access to care
Synovec et al., 2020<br><br>United States	Observational<br><br>To evaluate the integration of occupational therapy services in FQHC	- Age [Years]: 28-65<br>- Sex [Females]: 22.0%	- 51.0%	- The study setting was a FQHC, which provides comprehensive primary and integrated health care to individuals who are low income and/or experiencing homelessness	<u>Intervention:</u><br>- The occupational therapy intervention focused on participant goal attainment identified using the Canadian Occupational Performance Measure<br>- The intervention sessions consisted of preparatory methods (cognitive skill development), purposeful activity (time management and organization, goal setting), and occupation-based activity (meal preparation, home management)<br><br><u>Rehabilitation Team:</u><br>- Multidisciplinary team including physicians, nurses, SWs, case managers and registered OTs<br><br><u>Outcome:</u><br>- Integration of full-time occupational therapy services increased client access to functional-based rehabilitation services that addressed physical and behavioural health<br>- Clients demonstrated improvement towards functional goals using client-centred goal setting processes and interventions	NR
Witbeck et al., 1999	Observational<br><br>To determine whether	- Age [Mean ± SD]: 45.7 ± 9.9<br>- Race/Ethnicity: White (90.0%), African American (10%)	- 3 of the 18 participants had a hx of TBI	- 16 of the 18 participants were homeless	<u>Intervention:</u><br>- Emergency Services Outreach Program: Provided intensive, community case management services to engage Frequent	NR

Study (Author, Year, Country)	Study Design & Objective	Study Sample[a]	TBI Status	Homelessness Status	Rehabilitation Intervention, Team, Outcome	TBI-Specific Facilitators, Barriers, Gaps
United States	Emergency Services Outreach Program improved functional stability and decreased use of emergency services in Frequent Utilizers of Emergency Services				Utilizers of Emergency Services in ongoing tx and decrease emergency ambulatory use<br>- Case manager assisted in accessing resources, assessing and caring of MHSU, referring to appropriate tx settings, assisting in patient sobriety, and housing support and maintenance<br><br><u>Rehabilitation Team:</u><br>- Case Manager<br><br><u>Outcome:</u><br>- Individuals receiving Emergency Services Outreach Program services had a significant decrease in emergency ambulance use (58% decrease in monthly ambulance use and no change in the control group)	

Notes:

[a] Study sample's characteristics are reported as described in the manuscript; for example, if the study used the term "gender" but described their participants as "males" or "females", this was reported as "Gender [Males]" in the table

ACT: Assertive Community Treatment; **B/t:** Between; **FQHC:** Federally Qualified Health Centre; **GAS:** Goal Attainment Scales; **HF:** Housing First; **Hx:** History; **ICM:** Intensive Case Management; **MHSU:** Mental Health and/or Substance Use; **Mos:** Months; **NR:** Not reported; **OSU TBI- ID:** Ohio State University Traumatic Brain Injury Identification Method**; OTs:** Occupational Therapists; **QoL:** Quality of Life; **RCT:** Randomized Control Trial; **SWs:** Social Workers; **SUD:** Substance Use Disorder; **Sxs:** Symptoms; **TAU:** Treatment as Usual; **TBI:** Traumatic Brain Injury; **Tx:** Treatment; **VR:** Vocational Rehabilitation; **w/:** with; **WHO-DAS:** World Health Organization Disability Assessment Schedule; **Yr(s):** Year(s)
