# Supplementary material for: Rehabilitation among individuals experiencing homelessness and traumatic brain injury: A scoping review
Source: Front Med (Lausanne). 2022 Nov 11;9:916602. doi: 10.3389/fmed.2022.916602 (PMC9692012; doi:10.3389/fmed.2022.916602)
Supplement: Supplementary File 3 — Quality appraisal. [file Data_Sheet_3.pdf]

Quality Appraisal for Controlled Interventions (N=6)						
Authors	Adair et al.	Chung et al.	Kozloff et al.	Mejia-Lancheros et al.	Georing et al.	Stergiopoulos et al.
Year	2017	2018	2016	2020	2014	2014
Title	Outcome trajectories among homeless individuals with mental disorders in a multisite randomized controlled trial of housing first	Housing first for older homeless adults with mental illness: A subgroup analysis of the At Home/Chez Soi randomized controlled trial	The unique needs of homeless youths with mental illness: Baseline findings from a housing first trial	Effect of housing first on violence-related TBI in adults with experiences of homelessness and mental illness: Findings from the At Home/Chez Soi RCT Toronto site	National final report: Cross-site At Home/Chez Soi Project	Toronto final report: At Home/Chez Soi Project
**CRITERIA**						
1. Was the study described as randomized, a randomized trial, a randomized clinical trial, or an RCT?	Yes	Yes	Yes	Yes	Yes	Yes
2. Was the method of randomization adequate (i.e., use of randomly generated assignment)?	CD	Yes	Yes	Yes	NR	NR
3. Was the treatment allocation concealed (so that assignments could not be predicted)?	NR	Yes	NR	No	No	NR
4. Were study participants and providers blinded to treatment group assignment?	NR	Yes	NR	No	NR	NR
5. Were the people assessing the outcomes blinded to the participants' group assignments?	NR	Yes	NR	No	NR	NR
6. Were the groups similar at baseline on important characteristics that could affect outcomes (e.g., demographics, risk factors, co-morbid conditions)?	NR	No	NR	Yes	Yes	CD
7. Was the overall drop-out rate from the study at endpoint 20% or lower of the number allocated to treatment?	NR	Yes	NR	NR	NR	NR
8. Was the differential drop-out rate (between treatment groups) at endpoint 15 percentage points or lower?	NR	No	NR	NR	NR	NR
9. Was there high adherence to the intervention protocols for each treatment group?	Yes	NR	NR	NR	NR	NR
10. Were other interventions avoided or similar in the groups (e.g., similar background treatments)?	NR	NR	Yes	NR	Yes	NR
11. Were outcomes assessed using valid and reliable measures, implemented consistently across all study participants?	Yes	Yes	Yes	Yes	Yes	Yes
12. Did the authors report that the sample size was sufficiently large to be able to detect a difference in the main outcome between groups with at least 80% power?	NR	NR	NR	Yes	NR	NR
13. Were outcomes reported or subgroups analyzed prespecified (i.e., identified before analyses were conducted)?	Yes	Yes	Yes	Yes	Yes	Yes
14. Were all randomized participants analyzed in the group to which they were originally assigned, i.e., did they use an intention-to-treat analysis?	NR	Yes	NR	Yes	NR	NR
**Response Options:** Yes, No, CD (Cannot Determine), NR (Not Reported), or NA (Not Applicable)						

**Quality Appraisal for Observational Cohort Study or Cross-Sectional Studies (N=8)**								
**Authors**	Fine et al.	Gargaro et al.	Langi et al.	McHugo et al.	Schiff, JW.	Synovec and Berry	Synovec et al.	Witbeck et al.
**Year**	2021	2016	2017	2021	2014	2020	2020	2000
**Title**	Experience with a mobile addiction program among people experiencing homelessness	Brain injury in persons with serious mental illness who have a history of chronic homelessness: Could this impact how services are delivered?	Toward a successful vocational rehabilitation in adults with disabilities: Does residential arrangement matter?	Algorithm-drive substance use disorder treatment for inner-city clients with serious mental illness and multiple impairments	Comparison of four Housing First programs	Addressing brain injury in health care for the homeless settings: A pilot model for provider training	OT in integrated primary care: Addressing the needs of individuals experiencing homelessness	Emergency room outreach to chronically addicted individuals: A pilot study
**CRITERIA**								
1. Was the research question or objective in this paper clearly stated?	Yes	Yes	Yes	Yes	Yes	No	Yes	Yes
2. Was the study population clearly specified and defined?	Yes	Yes	Yes	Yes	Yes	Yes	Yes	Yes
3. Was the participation rate of eligible persons at least 50%?	Yes	Yes	Yes	Yes	NR	CD	Yes	Yes
4. Were all the subjects selected or recruited from the same or similar populations (including the same time period)? Were inclusion and exclusion criteria for being in the study prespecified and applied uniformly to all participants?	Yes	Yes	Yes	Yes	Yes	Yes	Yes	Yes
5. Was a sample size justification, power description, or variance and effect estimates provided?	NR	NR	NR	NR	NR	NR	NR	NR
6. For the analyses in this paper, were the exposure(s) of interest measured prior to the outcome(s) being measured?	Yes	Yes	Yes	Yes	Yes	NR	Yes	Yes
7. Was the timeframe sufficient so that one could reasonably expect to see an association between exposure and outcome if it existed?	CD	CD	NR	NR	NR	NR	NR	NR
8. For exposures that can vary in amount or level, did the study examine different levels of the exposure as related to the outcome (e.g., categories of exposure, or exposure measured as continuous variable)?	NA	NA	NA	NA	NA	CD	Yes	NA
9. Were the exposure measures (independent variables) clearly defined, valid, reliable, and implemented consistently across all study participants?	Yes	Yes	Yes	Yes	NR	NR	Yes	Yes
10. Was the exposure(s) assessed more than once over time?	No	No	No	No	No	No	Yes	No
11. Were the outcome measures (dependent variables) clearly defined, valid, reliable, and implemented consistently across all study participants?	Yes	Yes	Yes	Yes	Yes	No	Yes	Yes
12. Were the outcome assessors blinded to the exposure status of participants?	NA	No	No	No	No	No	No	No
13. Was loss to follow-up after baseline 20% or less?	NA	NA	NA	No	NR	No	NR	NR
14. Were key potential confounding variables measured and adjusted statistically for their impact on the relationship between exposure(s) and outcome(s)?	NA	NA	Yes	Yes	NA	NA	NR	NR
**Response Options:** Yes, No, CD (Cannot Determine), NR (Not Reported), or NA (Not Applicable)								

Quality Appraisal for Before-After (Pre-Post) Studies with no Control Grp (N=2)		
**Authors**	Bean et al.	Gutman et al.
**Year**	2013	2008
**Title**	The impact of housing first and peer support on people who are medically vulnerable and homeless	Enhancing independence in women experiencing domestic violence and possible brain injury
**CRITERIA**		
1. Was the study question or objective clearly stated?	Yes	Yes
2. Were eligibility/selection criteria for the study population prespecified and clearly described?	Yes	Yes
3. Were the participants in the study representative of those who would be eligible for the test/service/intervention in the general or clinical population of interest?	NR	NR
4. Were all eligible participants that met the prespecified entry criteria enrolled?	NR	No
5. Was the sample size sufficiently large to provide confidence in the findings?	NR	NR
6. Was the test/service/intervention clearly described and delivered consistently across the study population?	NR	Yes
7. Were the outcome measures prespecified, clearly defined, valid, reliable, and assessed consistently across all study participants?	Yes	Yes
8. Were the people assessing the outcomes blinded to the participants' exposures/interventions?	NR	NR
9. Was the loss to follow-up after baseline 20% or less? Were those lost to follow-up accounted for in the analysis?	No	NR
10. Did the statistical methods examine changes in outcome measures from before to after the intervention? Were statistical tests done that provided p values for the pre-to-post changes?	Yes	No
11. Were outcome measures of interest taken multiple times before the intervention and multiple times after the intervention (i.e., did they use an interrupted time-series design)?	No	No
12. If the intervention was conducted at a group level (e.g., a whole hospital, a community, etc.) did the statistical analysis take into account the use of individual-level data to determine effects at the group level?	No	NA
**Response Options:** Yes, No, CD (Cannot Determine), NR (Not Reported), or NA (Not Applicable)		

**Quality Appraisal for Case Studies/Case Series Studies (N=2)**		
**Authors**	Brocht et al.	Witbeck et al.
**Year**	2020	2000
**Title**	A clinical description of strategies to address TBI experienced by homeless patients at Baltimore's medical respite program	Emergency room outreach to chronically addicted individuals: A pilot study
**<u>CRITERIA</u>**		
1. Was the study question or objective clearly stated?	Yes	Yes
2. Was the study population clearly and fully described, including a case definition?	Yes	Yes
3. Were the cases consecutive?	CD	CD
4. Were the subjects comparable?	CD	CD
5. Was the intervention clearly described?	Yes	Yes
6. Were the outcome measures clearly defined, valid, reliable, and implemented consistently across all study participants?	Yes	Yes
7. Was the length of follow-up adequate?	CD	CD
8. Were the statistical methods well-described?	NR	NR
9. Were the results well-described?	Yes	Yes
**Response Options:** Yes, No, CD (Cannot Determine), NR (Not Reported), or NA (Not Applicable)		

Quality Appraisal for Qualitative Studies (N=2)		
**Authors**	Merryman et al.	Schiff, JW.
**Year**	2019	2014
**Title**	Integrated care: Provider referrer perceptions of OT services for homeless adults in an integrated primary care setting	Comparison of four Housing First programs
**CRITERIA**		
1. Was there a clear statement of the aims of the research?	Yes	Yes
2. Is a qualitative methodology appropriate?	Yes	Yes
3. Was the research design appropriate to address the aims of the research?	Yes	Yes
4. Was the recruitment strategy appropriate to the aims of the research?	No	Yes
5. Was the data collected in a way that addressed the research issue?	Yes	Yes
6. Has the relationship between researcher and participants been adequately considered?	No	Can't Tell
7. Have ethical issues been taken into consideration?	Yes	Yes
8. Was the data analysis sufficiently rigorous?	Yes	Yes
9. Is there a clear statement of findings?	Yes	Yes
10. How valuable is the research?	Yes	Yes
**Response Options:** Yes, No, Can't Tell		
